# Potent Platinum(IV) Prodrugs That Incorporate a Biotin Moiety to Selectively Target Cancer Cells

**DOI:** 10.3390/pharmaceutics14122780

**Published:** 2022-12-12

**Authors:** Aleen Khoury, Jennette A. Sakoff, Jayne Gilbert, Shawan Karan, Christopher P. Gordon, Janice R. Aldrich-Wright

**Affiliations:** 1 School of Science, Western Sydney University, Locked Bag 1797, Penrith South DC, NSW 2751, Australia; 2 Calvary Mater Hospital, Waratah, NSW 2298, Australia; 3 Teaching and Research Technical Services, Western Sydney University, Locked Bag 1797, Penrith South DC, NSW 2751, Australia

**Keywords:** 56MESS, biotin, cancer, cellular accumulation, cytotoxicity, lipophilicity, platinum(IV), prodrugs, receptor target, selectivity

## Abstract

Four platinum(IV) prodrugs incorporating a biotin moiety to selectively target cancer cells were synthesised, characterised, and their biological activity assessed. All complexes exhibited exceptional in vitro cytotoxicity against a panel of cancer cell lines, with [Pt(5,6-dimethyl-1,10-phenanthroline)(1*S*,2*S*-diaminocyclohexane)(biotin)(hydroxido)](NO_3_)_2_, (**2**) exhibiting the lowest GI_50_ of 4 nM in the prostate Du145 cancer cell line. Each complex displayed significantly enhanced activity compared to cisplatin, with **2** being 1000-fold more active in the HT29 colon cancer cell line. Against the MCF-7 breast cancer cell line, in which high levels of biotin receptors are expressed, **2**, [Pt(4,7-dimethoxy-1,10-phenanthroline)(1*S*,2*S*-diaminocyclohexane)(biotin)(hydroxido)](NO_3_)_2_, (**3**), and [Pt(5-methyl-1,10-phenanthroline)(1*S*,2*S*-diaminocyclohexane)(biotin)(hydroxido)](NO_3_)_2_, (**4**) exhibited enhanced activity compared to their platinum(II) cores, with **4** being 6-fold more active than its platinum(II) precursor. Furthermore, **3** exhibited 3-fold greater selectivity towards MCF-7 breast cancer cells compared to MCF10A breast healthy cells, and this was further confirmed by platinum uptake studies, which showed **3** to have almost 3-fold greater uptake in MCF-7 cells, compared to MCF10A cells. The results show that lipophilicity and selectivity both contributed to the cellular uptake of **1**–**4**; however, this was not always translated to the observed cytotoxicity.

## 1. Introduction

**Figure 1:**
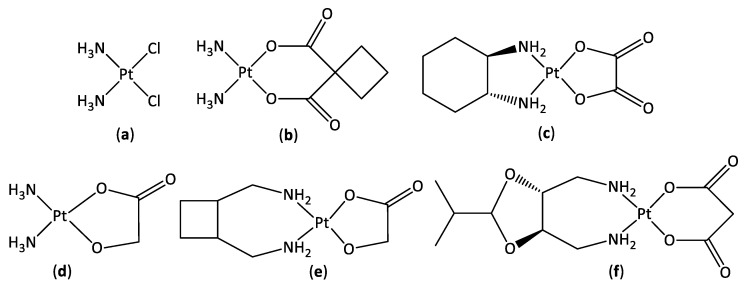
The chemical structures of cisplatin (**a**), carboplatin (**b**), oxaliplatin (**c**), nedaplatin (**d**), lobaplatin (**e**), and heptaplatin (**f**).

Cancer is the second leading cause of death worldwide, accounting for ~10 million deaths in 2020 [1]. Chemotherapy is one of the main forms of cancer treatment, with platinum-based drugs administered in ~50% of all chemotherapy treatments [2]. Platinum compounds approved by the FDA for cancer treatment worldwide include cisplatin and its derivatives, carboplatin and oxaliplatin (Figure 1), which are effective in the treatment of numerous cancer types [3,4,5]. The structural similarities of these platinum(II) complexes give rise to similar mechanisms of action where they bind covalently to DNA, distorting the double helix, which ultimately results in cell death [6,7]. Despite the great efficiency of these compounds against numerous cancers, their applicability is limited due to intrinsic and acquired drug resistance and toxic side-effects caused by reactions with off target molecules [8,9]. Third generation platinum(II) complexes have been developed and approved for regional use, including nedaplatin, lobaplatin, and heptaplatin, which are approved for clinical use in Japan, China, and the Republic of Korea, respectively (Figure 1) [10,11,12,13]. However, by employing the same square planar design, these second and third generation complexes elicit analogous mechanisms of action, with similar clinical challenges to cisplatin [11].

One way of addressing these limitations is by designing platinum compounds that deviate from the traditional structural design of cisplatin and its second and third generation derivatives. A promising group of unconventional platinum complexes of the type [Pt(P_L_)(A_L_)]^2+^, where P_L_ is a polyaromatic ligand such as 1,10-phenanthroline (Phen) and A_L_ is a chiral ancillary ligand, such as 1*S*,2*S*-diaminocyclohexane (SSDACH), exhibit in vitro nanomolar GI_50_ values across several cancer cell lines [14,15]. Dissimilar to cisplatin, this group of complexes exhibit multimodal mechanisms of action contributing to their cytotoxicity, where they were reported to alter the cytoskeletal environment, damage nuclear DNA, reduce the mitochondrial membrane potential, and induce epigenetic processes [16]. However, the noteworthy in vitro cytotoxicity of these compounds has not entirely translated in vivo, suggesting poor pharmacokinetics [17]. To address this, platinum(IV) complexes comprising two hydroxido axial ligands in the form [Pt(P_L_)(A_L_)(OH)_2_]^2+^ were created by oxidation, and have been reported to exhibit improved activity in vivo [18,19].

**Figure 2:**
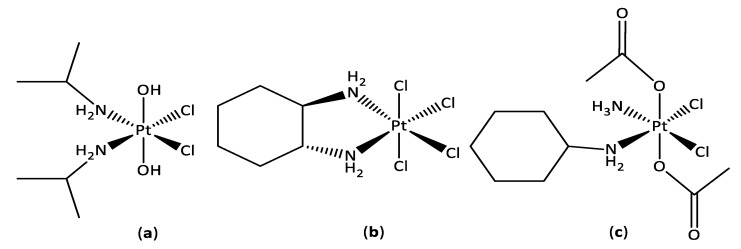
The chemical structures of platinum(IV) compounds iproplatin (**a**), tetraplatin (**b**), and satraplatin (**c**) that entered clinical trials.

Platinum(IV) compounds are more kinetically stable due to their low spin, d^6^ octahedral geometry, making them less susceptible to react with undesirable extracellular biomolecules, which is key in reducing side-effects [20,21,22]. The two additional ligands in the axial positions of platinum(IV) complexes allow for facile derivatisation to modify the pharmacological properties of the drug, including reduction potential, lipophilicity, bioactivity, and, more importantly, selectivity [23,24,25,26]. Platinum(IV) prodrugs have a single pharmacokinetic profile which has advantages over administering a cocktail of drugs in combination. Drug delivery is facilitated by reduction to platinum(II) with concomitant release of the axial ligands once inside the cell [27,28,29]. Three platinum(IV) complexes have entered clinical trials—iproplatin, tetraplatin, and satraplatin—however, iproplatin and tetraplatin were rejected due to severe neurotoxicity and lower activity than current platinum(II) drugs, respectively, which could be attributed to the simple axial ligands (Figure 2) [30]. Satraplatin in combination with prednisone completed phase III of clinical trials; however, satraplatin was not approved by the FDA due it not showing an overall improvement in survival [31,32,33,34,35,36].

To improve selectivity, targeting vectors have been tethered to many platinum(IV) prodrugs [9]. Suitable targets include receptors that are overexpressed on cancer cells, which can be exploited as biomarkers to selectively target and deliver the prodrug to cancer cells [13,37]. Due to the rapidly dividing nature of cancer cells, an increased level of vitamins, such as vitamin B_12_, folic acid, riboflavin, and biotin (vitamin H or B_7_) are necessary to sustain this rapid growth and are important for tumour proliferation [38,39]. Therefore, the receptors involved in the uptake of vitamins become overexpressed on the surface of cancer cells. Of these, biotin receptors, (known as sodium-dependent multivitamin transporters (SMVTs)), have been reported to be the most overexpressed vitamin receptors on cancer cells, although they have been investigated the least [38,39,40]. Some biotin-conjugated chemotherapeutics have been developed and have shown to selectivity target cancer cells [41,42,43,44,45].

**Figure 3:**
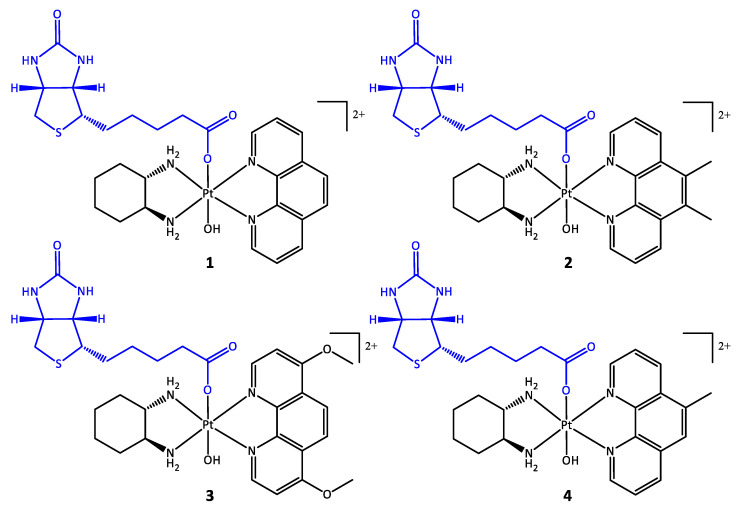
The chemical structures of complexes **1**–**4** comprised of biotin (blue) conjugated to different platinum cores (black) PHENSS (**1**), 56MESS (**2**), 47OMESS (**3**), and 5MESS (**4**).

To improve the selectivity of [Pt(P_L_)(A_L_)(OH)_2_]^2+^-type complexes towards cancer cells, we have developed a series of biotin conjugated platinum(IV) prodrugs of the type [Pt(P_L_)(A_L_)(biotin)(OH)]^2+^, where P_L_ = Phen or a substituted variant of Phen and A_L_ = SSDACH (Figure 3). These include: [Pt(1,10-phenthroline)(1*S*,2*S*-diaminocyclohexane)(biotin)(hydroxido)](NO_3_)_2_ (**1**), [Pt(5,6-dimethyl-1,10-phenanthroline)(1*S*,2*S*-diaminocyclohexane)(biotin)(hydroxido)](NO_3_)_2_ (**2**), [Pt(4,7-dimethoxy-1,10-phenanthroline)(1*S*,2*S*-diaminocyclohexane)(biotin)(hydroxido)](NO_3_)_2_ (**3**), and [Pt(5-methyl-1,10-phenanthroline)(1*S*,2*S*-diaminocyclohexane)(biotin)(hydroxido)](NO_3_)_2_ (**4**). Previous studies have explored the conjugation of COX inhibitors as well as long chain fatty acids to these types of platinum complexes and whilst they displayed promising results, they were not shown to exhibit selectivity towards cancer cells in vitro [24,46,47]. This is the first report of a receptor targeting moiety conjugated to these types of platinum complexes. Additionally, previous studies have utilised the platinum(II) cores **PHENSS(II)** and **56MESS(II)** in prodrugs, whereas here, we have developed a series of compounds with four different cores to determine how the core influences their activity (Figure 3). The complexes were fully characterised using a range of biophysical techniques, their lipophilicity was ascertained using RP-HPLC, and NMR spectroscopy was used to assess their reduction half-life in the presence of ascorbic acid. The in vitro cytotoxicity was determined using MTT assays against a group of cancerous cell lines and one non-cancerous cell line to assess their selective toxicity towards cancer cells. To further investigate their activity, ICP-MS was utilised to investigate the cellular accumulation in two breast cell lines: the cancerous MCF-7 cell line, which has a high level of biotin expression, and the non-cancerous MCF10A cell line, which has no biotin expression [45].

## 2. Materials and Methods

### 2.1. Materials

All reagents were used as received without further purification. MilliQ^TM^ water (Merck, Melbourne, Australia) was utilised, and organic solvents were of analytical grade. We acquired 1*S*,2*S*-Diaminocyclohexane (SSDACH, 98%), 4,7-dimethoxy-1,10-phenanthroline (47O_2_Me_2_Phen), 1,10-phenanthroline (Phen), 5-methyl-1,10-phenanthroline (5MePhen), 5,6-dimethyl-1,10-phenanthroline (56Me_2_Phen), biotin, silver nitrate, *N*-(3-Dimethylaminopropyl)-*N*’-ethylcarbodiimide hydrochloride (EDC-HCl), and *N*-hydroxysuccinimide (NHS) from Sigma-Aldrich, Sydney, NSW. Potassium tetrachloroplatinate (K_2_PtCl_4_) was obtained from Precious Metals Online. Potassium iodide (KI) was purchased from Merck, Melbourne, Australia. Hydrogen peroxide (H_2_O_2_, 30%) was obtained from VWR chemicals, Philadelphia, USA. Acetonitrile (ACN), dimethyl formamide (DMF), diethyl ether, methanol (MeOH), and dimethyl sulfoxide (DMSO) were purchased from Chem-Supply, Adelaide, Australia. Sep-Pak^®^ C18 columns were acquired from Waters, Australia. For ICP-MS experiments, the certified reference standard used was purchased from High-Purity standards, North Charleston, United States. Ultra-pure HNO_3_ (69%) was obtained from Choice Analytical, Sydney, Australia. Deuterated solvents used for NMR experiments (d_6_-dimethyl sulfoxide (DMSO-d_6_, 99.9%) and deuterium oxide (D_2_O, 99.9%) were acquired from Novachem, Melbourne, Australia.

### 2.2. Synthesis of [Pt(P_L_)(SSDACH)]Cl_2_ (PHENSS(II), 56MESS(II), 47OMESS(II), and 5MESS(II)) and [Pt(P_L_)(SSDACH)(OH)_2_](NO_3_)_2_ (PHENSS(IV), 56MESS(IV), 47OMESS(IV), and 5MESS(IV)) (Where P_L_ = Phen, 56Me_2_Phen, 47O_2_Me_2_Phen or 5MePhen)

The platinum(II) and platinum(IV) precursors were synthesised following previously published methods [18,46,48].

### 2.3. Synthesis of Biotin-NHS Ester

The synthesis of Biotin-NHS followed a method previously published [42]. Biotin (400 mg, 1.64 mmol), NHS (0.283 g, 2.46 mmol), and EDC-HCl (0.345 g, 1.80 mmol) were dissolved in anhydrous DMF (12 mL) and left to stir at room temperature overnight. The resulting solution was then added to ice-cold water (80 mL) forming a white precipitate. Vacuum filtration was used to collect the precipitate which was then washed with H_2_O (×2) and MeOH (×2). The biotin-NHS ester was used immediately for the next step (conjugated to platinum) or stored at −20 °C. Yield (463.9 mg, 83%). ^1^H NMR (400 MHz, DMSO-d_6_) δ (ppm): 6.42 (s, 1H), 6.37 (s, 1H), 4.31 (m, 1H), 4.15 (m, 1H), 3.11 (m, 1H), 2.82 (m, 5H), 2.68 (t, *J* = 7.34 Hz, 2H), 2.59 (d, *J* = 12.43 Hz, 1H), 1.66 (m, 3H), 1.46 (m, 3H).

### 2.4. Synthesis of [Pt(P_L_)(SSDACH)(Biotin)(OH)](NO_3_)_2_ (P_L_ = Phen (***1***), 56Me_2_Phen (***2***), 47O_2_Me_2_Phen (***3***), or 5MePhen (***4***))

[Pt(P_L_)(SSDACH)(OH)_2_](NO_3_)_2_ (200 mg) and 2 mol. equiv. of Biotin-NHS ester were dissolved in DMSO (1.5–2 mL) and stirred in the dark at 40 °C overnight. This produced a golden-green solution which was diluted with MeOH (6 mL) and precipitated with ether (~40 mL). The resulting white precipitate was collected by vacuum filtration and washed with ether. To purify these complexes, a flash chromatography system was employed. Complexes were dissolved in minimal water then eluted through a C18 column over different H_2_O/MeOH gradients at a flowrate of 4 mL/min (outlined in Table S1).

#### 2.4.1. [Pt(Phen)(SSDACH)(Biotin)(OH)](NO_3_)_2_ (**1**)

Yield (178.1 mg, 66%). Electronic spectrum λ_max_ nm (ε/mol^−1^ dm^3^ cm^−1^, water): 204 (70,400 ± 180), 279 (25,300 ± 70), 306 (6200 ± 85). CD spectrum λ_max_ nm (mdeg mol L^−1^, water): 212 (−2.8), 276 (−0.4). ^1^H NMR (400 MHz, D_2_O) δ (ppm): 9.34 (d, *J* = 5.46 Hz, 1H), 9.26 (d, *J* = 5.51 Hz, 1H), 9.01 (dd, *J*_1_ = 8.26 Hz, *J*_2_ = 1.85 Hz, 2H), 8.34 (s, 2H), 8.27 (dd, *J*_1_ = 8.07 Hz, *J*_2_ = 5.71 Hz, 2H), 4.43 (dd, *J*_1_ = 7.90 Hz, *J*_2_ = 4.83 Hz, 1H), 4.01 (dd, *J*_1_ = 7.96 Hz, *J*_2_ = 4.55 Hz, 1H), 3.16 (m, 2H), 2.78 (m, 2H), 2.61 (d, *J* = 13.11 Hz, 1H), 2.39 (m, 2H), 2.06 (m, 2H), 1.68 (m, 4H), 1.30 (m, 2H), 1.17 (m, 2H), 1.04 (m, 1H), 0.91 (m, 1H), 0.60 (m, 1H), 0.46 (m, 1H). ^1^H-^195^Pt HMQC (400/86 MHz, D_2_O): δ 9.36, 9.25, 8.26/539. ^195^Pt NMR (86 MHz, D_2_O): δ 539 ppm. HPLC *t*_R_: 5.7 min. HRMS-ESI: Calc. [M-H]^+^ *m*/*z* = 748.2245, found = 748.2262.

#### 2.4.2. [Pt(56Me_2_Phen)(SSDACH)(Biotin)(OH)](NO_3_)_2_ (**2**)

Yield (170.9 mg, 64%). Electronic spectrum λ_max_ nm (ε/mol^−1^ dm^3^ cm^−1^, water): 206 (75,900 ± 360), 290 (26,800 ± 170), 318 (6700 ± 30). CD spectrum λ_max_ nm (mdeg mol L^−1^, water): 212 (−2.6), 243 (−0.6), 256 (0.1), 286 (−0.6). ^1^H NMR (400 MHz, D_2_O) δ (ppm): 9.29 (d, *J* = 5.48 Hz, 1H), 9.19 (m, 3H), 8.24 (m, 2H), 4.39 (dd, *J*_1_ = 7.85 Hz, *J*_2_ = 4.72 Hz, 1H), 3.93 (dd, *J*_1_ = 7.88 Hz, *J*_2_ = 4.48 Hz, 1H), 3.14 (m, 2H), 2.83 (s, 6H), 2.70 (m, 2H), 2.55 (d, *J* = 12.95 Hz, 1H), 2.38 (m, 2H), 2.15 (m, 1H), 2.00 (m, 1H), 1.67 (m, 4H), 1.29 (m, 2H), 1.11 (m, 2H), 1.01 (m, 1H), 0.73 (m, 1H), 0.37 (m, 1H), 0.23 (m, 1H). ^1^H-^195^Pt HMQC (400/86 MHz, D_2_O): δ 9.30, 9.17, 8.25/527. ^195^Pt NMR (86 MHz, D_2_O): δ 526 ppm. HPLC *t*_R_: 6.2 min. HRMS-ESI: Calc. [M-H]^+^ *m*/*z* = 776.2558, found = 776.2574.

#### 2.4.3. [Pt(47O_2_Me_2_Phen)(SSDACH)(Biotin)(OH)](NO_3_)_2_ (**3**)

Yield (163.7 mg, 62%). Electronic spectrum λ_max_ nm (ε/mol^−1^ dm^3^ cm^−1^, water): 194 (110,400 ± 190), 212 (103,200 ± 130), 267 (60,100 ± 130), 280 (38,100 ± 320), 347 (8200 ± 15). CD spectrum λ_max_ nm (mdeg mol L^−1^, water): 214 (−2.7), 247 (0.1), 268 (−0.5), 305 (0.5). ^1^H NMR (400 MHz, D_2_O) δ (ppm): 9.08 (d, *J* = 6.74 Hz, 1H), 8.99 (d, *J* = 6.76, 1H), 8.38 (s, 2H), 7.63 (dd, *J*_1_ = 6.84 Hz, *J*_2_ = 2.04 Hz, 2H), 4.43 (dd, *J*_1_ = 7.86 Hz, *J*_2_ = 4.84 Hz, 1H), 4.31 (s, 6H), 3.97 (dd, *J*_1_ = 7.88 Hz, *J*_2_ = 4.53 Hz, 1H), 3.11 (m, 2H), 2.67 (m, 3H), 2.37 (m, 2H), 2.07 (m, 2H), 1.67 (m, 4H), 1.28 (m, 2H), 1.14 (m, 2H), 1.01 (m, 1H), 0.84 (m, 1H), 0.45 (m, 1H), 0.33 (m, 1H). ^1^H-^195^Pt HMQC (400/86 MHz, D_2_O): δ 9.08, 8.97, 7.63/590. ^195^Pt NMR (86 MHz, D_2_O): δ 590 ppm. HPLC *t*_R_: 6.5 min. HRMS-ESI: Calc. [M-H]^+^ *m*/*z* = 808.2456, found = 808.2479.

#### 2.4.4. [Pt(5MePhen)(SSDACH)(Biotin)(OH)](NO_3_)_2_ (**4**)

Yield (174.5 mg, 65%). Electronic spectrum λ_max_ nm (ε/mol^−1^ dm^3^ cm^−1^, water): 206 (72,400 ± 310), 284 (25,800 ± 160), 312 (6500 ± 50). CD spectrum λ_max_ nm (mdeg mol L^−1^, water): 212 (−1.8), 242 (−0.3), 251 (0.3), 284 (−0.3). ^1^H NMR (400 MHz, D_2_O) δ (ppm): 9.35 (d, *J* = 5.61 Hz, 1H), 9.25 (t, *J* = 4.83 Hz, 1H), 9.17 (m, 1H), 8.96 (m, 1H), 8.29 (m, 1H), 8.20 (m, 1H), 8.15 (s, 1H), 4.42 (dd, *J*_1_ = 7.83 Hz, *J*_2_ = 4.97 Hz, 1H), 3.97 (m, 1H), 3.16 (m, 2H), 2.90 (s, 3H), 2.74 (m, 2H), 2.59 (m, 1H), 2.38 (m, 2H), 2.12 (m, 1H), 2.01 (m, 1H), 1.68 (m, 4H), 1.29 (m, 2H), 1.14 (m, 2H), 1.02 (m, 1H), 0.81 (m, 1H), 0.49 (m, 1H), 0.33 (m, 1H). ^1^H-^195^Pt HMQC (400/86 MHz, D_2_O): δ 9.37, 9.26, 9.14/540. ^195^Pt NMR (86 MHz, D_2_O): δ 540 ppm. HPLC *t*_R_: 5.9 min. HRMS-ESI: Calc. [M-H]^+^ *m*/*z* = 762.2401, found = 762.2419.

### 2.5. NMR Spectroscopy

We obtained 1D and 2D NMR spectra using a Bruker Avance (Melbourne, Australia) 400 MHz spectrometer. All spectral data were acquired at 298 K and samples were prepared in either D_2_O or DMSO-d_6_. For ^1^H NMR spectra, 65,536 data points were obtained, with a spectral width of 8251 Hz. COSY spectra were acquired using a spectral width of 4085 Hz for both ^1^H nuclei (F1 and F2 dimensions), with 256 data points for F1 and 2048 data points for F2. ^195^Pt NMR spectra were recorded with 674 data points and a spectral width of 85,470 Hz. ^1^H-^195^Pt HMQC spectra were obtained using a spectral width of 215,156 Hz and 256 data points for the ^195^Pt nucleus (F1 dimension) and a spectral width of 4808 Hz and 2048 data points for the ^1^H nucleus (F2 dimension). The chemical shifts are reported in parts per million (ppm) and the *J* coupling constants are reported in Hz.

### 2.6. Flash Chromatography

A Biotage Isolera^TM^ One flash chromatography system (Shimadzu, Sydney, Australia) was used to purify the platinum complexes (**1**–**4**). Samples were dissolved in minimal water and eluted through a Biotage SNAP KP-C18-HS 12 g cartridge (Shimadzu, Sydney, Australia) using varying H_2_O/MeOH gradients at 4 mL/min (Table S1). Sample absorbances were monitored at wavelengths 230 and 280 nm, using a photodiode array (PDA).

### 2.7. Ultraviolet (UV) Absorption Spectroscopy

An Agilent Cary 3500 UV-Vis spectrophotometer (Melbourne, Australia) was used to obtain UV absorbance spectra. Samples were prepared in water at room temperature. A 1 cm quartz cuvette was used to collect data from 190–400 nm, with water as the reference. To determine the extinction coefficients, a concentrated stock solution of each complex (1 mM) was initially prepared in water and aliquoted (2–5 µL) into a cuvette containing water (3000 µL). Experiments were repeated in triplicate and the average extinction coefficients were calculated.

### 2.8. Circular Dichroism (CD) Spectroscopy

Circular dichroism (CD) spectra were obtained using a Jasco J-810 CD spectropolarimeter (Easton, United States) in the range 200–400 nm. All samples were prepared at room temperature in water in a 1 cm cylindrical quartz cuvette. Spectra were obtained over 50 accumulations using a scan speed of 100 nm/min, a spectral bandwidth of 1 nm, a response of 1 s, a data pitch of 1 nm, and a sensitivity setting of 100 mdeg, with nitrogen gas flowing at 6 L/min. A water baseline was subtracted from each spectrum and HT levels remained below 450 V for all samples.

### 2.9. Mass Spectrometry (MS)

A Waters (Sydney, Australia) SYNAPT G2-S quadruple time-of-flight (QTOF) HDMS equipped with an ESI source was used to obtain spectra in positive ion mode. All samples were prepared in water at a concentration of 10–100 ng/mL. Spectra were acquired in the range *m*/*z* = 50–1200 Da using a capillary voltage ranging from 1–2.0 kV.

### 2.10. High Performance Liquid Chromatography (HPLC) and Lipophilicity Measurements

HPLC experiments were carried out using an Agilent Technologies (Melbourne, Australia) 1260 Infinity instrument, equipped with a Phenomenex (Sydney, Australia) Onyx^TM^ Monolithic C18 reverse phase column (100 × 4.6 mm, 130 Å). The mobile phase was made up of 0.06% TFA in water (solvent A) and 0.06% TFA in an acetonitrile:water (9:1) mixture (solvent B). Samples were eluted over a gradient of 0–100% (% B) for 15 min, at a flow rate of 1 mL/min, with a sample injection volume of 10 µL. Eluting peaks were detected using a PDA at 254 nm.

For lipophilicity experiments, RP-HPLC was utilised to determine the $\log k_{w}$ of each compound, similarly to what has been previously published for similar complexes [46]. Samples were eluted at various isocratic percentages, ranging from 18–30% solvent B. To determine the dead volume of the column, potassium iodide was used as an external dead volume marker. The retention time of each sample in each isocratic run ($t_{r}$) and the dead volume time ($t_{0}$) were both substituted in Equation (1) to calculate the capacity factor (k).

(1) $$k=\frac{t_{r}-t_{0}}{t_{0}}$$

Following this, logk was plotted against the percentage of ACN. For each complex, a minimum of four concentrations of ACN were used. This produced a linear graph with the equation in the form:

(2) $$\log k=S\varphi +\log k_{w}$$

where S is the slope, φ is the percentage of ACN, and $\log k_{w}$ is the *y*-intercept, which represents the capacity factor of the compound in 100% water.

### 2.11. In Vitro Cytotoxicity Assay

The in vitro cytotoxicity assays were undertaken at Calvary Mater Hospital, Waratah, NSW, Australia, following a method previously published in the literature [47]. A stock solution for each compound was prepared in DMSO (30 mM) and stored at −20 °C. The cell lines used in the study include HT29 colon, U87 glioblastoma, MCF-7 breast, A2780 ovarian, H460 lung, A431 skin, Du145 prostate, BE2-C neuroblastoma, SJ-G2 glioblastoma, MIA pancreas, and cisplatin-resistant ADDP ovarian (subclone of A2780) cancer cells, as well as the non-cancerous-derived MCF10A breast cell line. The cancer cell lines were cultured in a humidified atmosphere 5% CO_2_ at 37 °C and maintained in Dulbecco’s modified Eagle’s medium (Trace Biosciences, Sydney, Australia), supplemented with 10% foetal bovine serum, sodium bicarbonate (10 mM), penicillin (100 IU/mL), streptomycin (100 µg/mL), and glutamine (4 mM). The non-cancerous MCF10A cell line was maintained in DMEM:F12 (1:1) cell culture media, with 5% heat inactivated horse serum, supplemented with penicillin (50 IU/mL), streptomycin (50 µg/mL), glutamine (2 mM), HEPES (20 mM), epidermal growth factor (20 ng/mL), hydrocortisone (500 ng/mL), cholera toxin (100 ng/mL), and insulin (10 µg/mL). Cells were plated in duplicate in medium (100 µL) at a density of 2500–4000 cells per well in 96-well plates. On day 0, (24 h after plating) when the cells were in logarithmic growth, medium (100 μL) with or without the test agent was added to each well. The growth inhibitory effects were evaluated after 72 h drug exposure, using the MTT (3-(4,5-dimethylthiazol-2-yl)-2,5-diphenyltetrazolium bromide) assay, with the absorbance read at 540 nm. An eight-point dose–response curve was produced, which was used to determine the concentration at which growth was inhibited by 50% (GI_50_). This calculation was based on the difference between the optical density values on day 0 and those at the end of drug exposure. The GI_50_ values for cisplatin, carboplatin, oxaliplatin, **PHENSS(II)**, **56MESS(II)**, **PHENSS(IV)**, and **56MESS(IV)** were taken from the literature, where the same method was used [18,46,47]. Experiments were conducted on three separate occasions and GI_50_ values were reported as the mean and standard error of the mean.

### 2.12. Cellular Accumulation

Cellular uptake studies were carried out in MCF-7 (breast cancer) and MCF10A (healthy breast) cell lines. Reported in Table S2 are the numbers of cells per well that were seeded onto 12-well plates for each replicate and cell line. After incubating overnight at 37 °C, the cell culture medium was replaced with fresh media containing the test compounds at concentrations of 1.0 and 0.1 µM. After incubating for 4 h, the medium was removed, the cells were washed with PBS twice, and then incubated with trypsin solution until the cells dissociated from the plate. The harvested cells were then placed into an Eppendorf tube and PBS was added to a volume of 1 mL. Cell numbers were determined using trypan blue staining. Cell pellets were obtained after centrifugation and the cells were dispersed in 50 µL of MilliQ™ water and stored immediately at −20 °C until platinum analysis was conducted. These experiments were repeated in triplicate on three separate occasions.

### 2.13. Platinum Analysis

Sixty-nine percent HNO_3_ (50 µL) was added to each sample and digested at 80 °C for 1.5 h, before being taken up to 1 mL with H_2_O. The platinum analysis was conducted using a PerkinElmer (Melbourne, Australia) NexION™ 300X inductively coupled plasma mass spectrometer (ICP-MS). A four-point calibration curve was produced by serial dilution of a certified platinum reference standard. The calibration was linear over the working range (*R*^2^ > 0.9999) and the most abundant platinum isotope (*m*/*z* = 195) was monitored. The average values for three independent experiments were reported with standard deviations.

## 3. Results and Discussion

### 3.1. Synthesis and Characterisation

The synthesis of the platinum(II) (**PHENSS(II)**, **56MESS(II)**, **47OMESS(II)**, **and 5MESS(II)**) and di-hydroxido platinum(IV) (**PHENSS(IV)**, **56MESS(IV)**, **47OMESS(IV)**, **and 5MESS(IV)**) precursors were completed following previous methods with no modifications [18,46,48]. The NHS ester of biotin was initially synthesised, which was subsequently reacted with the corresponding di-hydroxido platinum(IV) complex in minimal DMSO. For the more lipophilic Phen derivatives, slightly more DMSO was used to ensure complete dissolution. ^195^Pt NMR experiments were used to monitor the formation of the mono-substituted complex from the di-hydroxido platinum(IV) complex in the reaction to mixture. In DMSO, a platinum chemical shift of ~330 ppm correlates with the unreacted di-hydroxido platinum(IV), while a platinum chemical shift of ~480 ppm corresponds to the mono-substituted platinum(IV) complexes of this type [24,48].

The first reaction was initially trialled using 2 molar equivalents of the biotin-NHS ester and left to stir at room temperature. After ~12 h, ^195^Pt NMR showed that a significant amount of di-hydroxido platinum(IV) remained unreacted in the solution. Consequently, it was heated to 45 °C, and was shown to fully react after another ~12 h. Thus, for the remaining complexes, rather than increasing the equivalence of the biotin, the reactions were heated to 45 °C since heat facilitated the reaction, and full conversion was achieved within 12 h. After full conversion to the mono-substituted complex, the solution was diluted with MeOH, and the product was precipitated by the addition of diethyl ether. All complexes were purified using flash chromatography to isolate the mono-substituted complex from any unreacted ligand or platinum. The resulting white precipitate was dissolved in water, which permitted the insoluble biotin to be filtered off before purification using flash chromatography. Complexes were eluted at a flow rate of 4 mL/min, using a C18 12 g cartridge and various H_2_O/MeOH gradients (Table S1).

We used 1D and 2D NMR spectroscopy to evaluate the structure and purity of each complex (Figures S1–S18). RP-HPLC was utilised to confirm the purity of each complex, with all chromatograms showing peak areas greater than 95% (Figures S19–S22). ESI-MS produced the correct mass peaks and expected isotopic distribution patterns for platinum complexes (Figures S23–S26). All characterisation data confirmed the successful synthesis and purification of each complex (Table S3).

### 3.2. NMR Spectroscopy

A combination of 1D (^1^H and ^195^Pt) and 2D (^1^H-^1^H COSY and ^1^H-^195^Pt HMQC) NMR experiments were undertaken to characterise each complex (Figures S1–S18). The NMR spectra of the platinum(II) and platinum(IV) precursors were each in agreement with published data [18,49]. In the ^1^H NMR spectra of **1**–**4**, the resonances arising from the P_L_ in the aromatic region and those observed in the aliphatic region corresponding to the A_L_ SSDACH were all consistent with previously published NMR elucidations [18,49].

**Figure 4:**
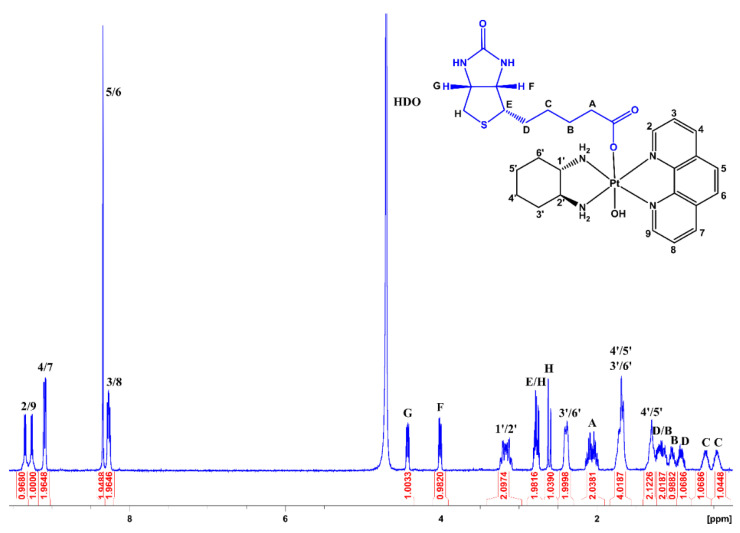
The ^1^H NMR spectrum of [Pt(Phen)(SSDACH)(Biotin)(OH)](NO_3_)_2_ (**1**) in D_2_O at 298 K. Insert: The chemical structure of **1** with assigned numbering/lettering.

Also depicted in the aliphatic region are the resonances from the axially bound biotin ligand, which displays resonances comparable to previous literature (Figures S3, S7, S11 and S15) [42]. In the ^1^H NMR spectrum of **1**, there were resonances at 4.43 and 4.01 ppm, each integrating for 1 proton (Figure 4). The resonance at 4.43 ppm was assigned to G, as this was coupled to two protons (at 2.78 and 2.61 ppm, assigned to protons H), whilst the resonance at 4.01 ppm was assigned to F, as this was only coupled to one resonance (2.78 ppm, assigned to E). In the COSY spectrum, E was also coupled to two resonances at 1.17 and 0.91 ppm, and thus assigned to the two protons at D. The resonances for D were also coupled to two additional resonances, at 0.60 and 0.46 ppm, corresponding to C. The protons at C were also each coupled to the resonances at 1.17 and 1.04 ppm, and therefore assigned to B, which was coupled to the multiplet integrating for two protons at 2.06 ppm. This multiplet was assigned to A, which exhibited a downfield chemical shift due to the electronegativity from the oxygens. The assignment for the protons of biotin in **2**–**4** followed the same rationale as **1** (Figures S7, S11 and S15).

The amine proton resonances were not observed in any of the spectra due to exchange with D_2_O. Since the platinum coordination sphere is sensitive to any changes, ^195^Pt and ^1^H-^195^Pt HMQC NMR experiments were undertaken to determine the platinum chemical shift and confirm the successful conjugation of biotin to the platinum centre (Figures S5, S6, S9, S10, S13, S14, S17 and S18). The di-hydroxido platinum(IV) precursors from this group exhibit platinum chemical shifts at ~400 ppm (in D_2_O), whilst the mono-substituted complexes experience a downfield chemical shift, towards ~530 ppm [46,48]. Complexes **1**, **2**, and **4** exhibited platinum chemical shifts at ~530 ppm, consistent with similar mono-substituted complexes; however, **3**, with the coordinated P_L_ 4,7-dimethoxy-1,10-phenanthroline, exhibited a further downfield shift at ~590 ppm. This increased downfield shift was also exhibited by its di-hydroxido platinum(IV) precursor and is due to the electronegativity of the methoxy groups on the phenanthroline [49]. The NMR characterisation confirmed the successful conjugation of biotin to platinum for each complex.

### 3.3. Electronic Spectra

**Figure 5:**
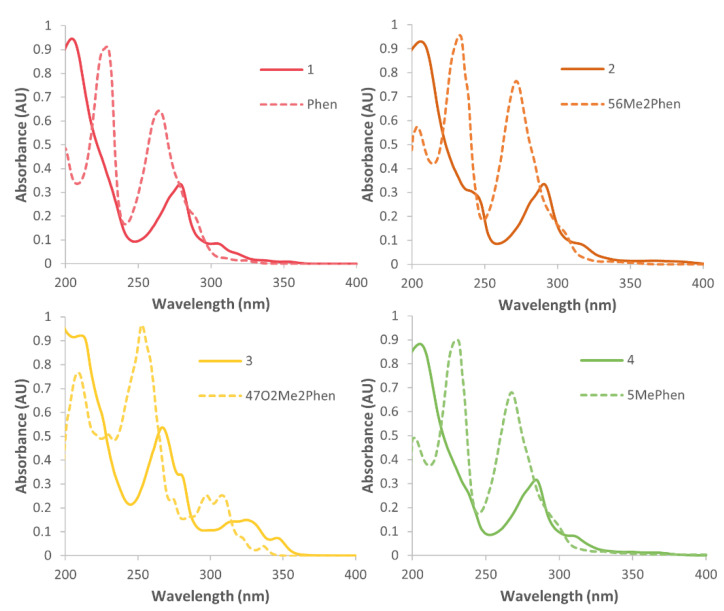
The UV spectra of complexes **1**–**4** overlaid with their corresponding P_L_, in water.

UV experiments were completed for **1**–**4** and their molar extinction coefficients were calculated (Table S3 and Figures S27–S30). The spectra displayed the expected π–π* transitions arising from the 1,10-phenthanroline and metal-to-ligand charge transfer (MLCT) interactions [18]. There were two distinct absorbance bands produced by each complex, with additional weaker bands. Complexes **1**, **2**, and **4** each displayed a strong absorbance band at ~205 nm; however, **3**, with the 4,7-dimethoxy substituent, exhibited a blue-shift of 11 nm compared to them, absorbing at 194 nm (Figure 5) The second strong absorbance band was present in the spectra of **1**, **2**, and **4** at ~280 nm, and it was observed that the addition of methyl groups on the P_L_ resulted in this band being red-shifted. For **1**, with the Phen substituent, this absorbance band was observed at 279 nm. The addition of one methyl group in **4** resulted in a red-shift of 5 nm, absorbing at 284 nm, and the addition of a second methyl group in **2** resulted in another red-shift, of 6 nm, absorbing at 290 nm. The red-shift caused by the methyl groups on the Phen is consistent with that observed by similar compounds published previously [18,49,50]. In contrast, the methoxy groups present in **3** resulted in a blue-shift in this region, showing it absorbing strongly at 267 nm. The spectra of **1**, **2**, and **4** also displayed a weak absorption band at ~306 nm, which was red-shifted by 6 nm by the addition of each methyl group. The band in this region was not clearly defined for **3**. The spectrum of **3** also depicted additional peaks at 212, 280, and 347 nm, which are attributed to the substituents on the Phen, as these were also present in the spectrum of the 47O_2_Me_2_Phen ligand. A shoulder at ~240 nm was observed for each complex, which is attributed to the MLCT interactions, since it was not exhibited by the individual P_L_s.

**Figure 6:**
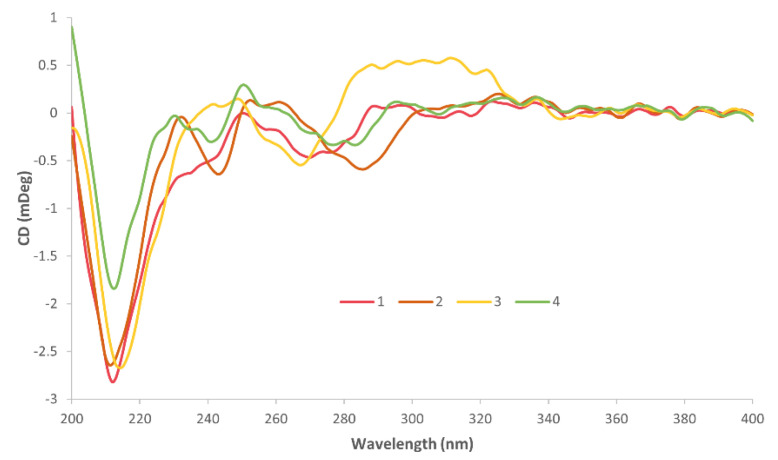
The CD spectra of **1**–**4** in water, over 50 accumulations.

CD spectroscopy was used to confirm the chirality of each complex (Figures S31–S34). The chirality of the A_L_ is important for the activity of these complexes since the SSDACH derivatives demonstrate superior activity to their RRDACH analogues, and thus it is vital to ensure that the chirality was conserved during synthesis [51,52]. As shown in Figure 6, the main similarity depicted in the CD spectra of **1**–**4** is the strong negative peak at ~212 nm. Complex **1**, with the P_L_ Phen, exhibited only one additional peak that was clearly defined at 276 nm. Complexes **4** and **2**, with the P_L_ comprising of one and two methyl substituents, respectively, exhibited comparable spectra, with both showing two additional negative peaks at ~242 and ~285 nm and one positive peak at ~254 nm. Complex **3**, with the P_L_ containing the methoxy groups exhibited distinctly different absorption patterns, showing one additional negative peak at 268 nm and two positive peaks at 247 and 305 nm. The general spectral pattern of **1**–**4** is consistent with previously published complexes that include the coordinated SSDACH and confirms that the chirality was retained during synthesis [18,49].

### 3.4. Reduction Studies via ^1^H NMR

**Figure 7:**
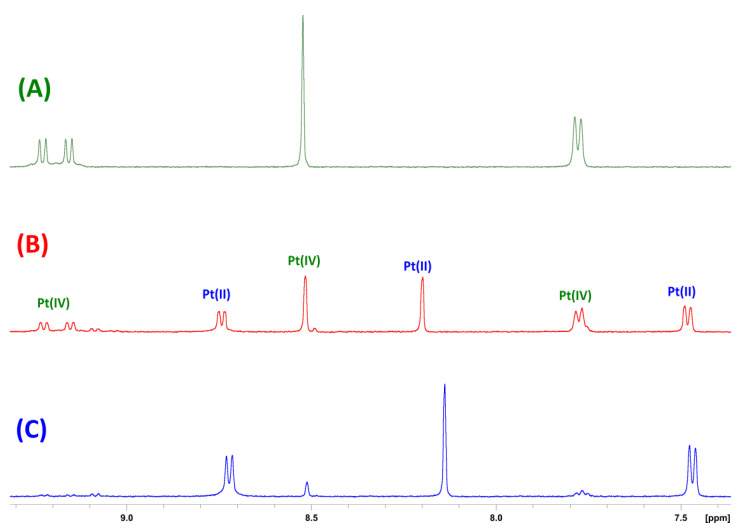
The ^1^H NMR spectra of the aromatic region of **3** with 10X PBS in D_2_O (5 mM) at 37 °C (**A**) before the addition of ascorbic acid; (**B**) after the addition of ascorbic acid (5 mM), showing the Pt(IV) and new Pt(II) resonances; (**C**) after reduction to Pt(II).

To confirm that the reduction of these prodrugs occurs and the axially bound biotin ligands are released from the cytotoxic platinum(II) core, reduction studies were undertaken using ^1^H NMR experiments for **3**. The complex was prepared in 5 mM D_2_O with 10X PBS. Ascorbic acid (5 mM) was added, and the reduction was monitored at 37 °C by ^1^H NMR experiments. Over time, the resonances corresponding to the platinum(IV) species decreased, while new resonances corresponding to the platinum(II) species increased (Figure 7). Subsequent ^195^Pt NMR experiments showed that the peak corresponding to the prodrug at 590 ppm had disappeared, and a new peak at -2801 ppm had developed, confirming reduction to its platinum(II) congener (Figures S35 and S36). The percentage of platinum(II) and platinum(IV) species were graphed over time and a half-life of ~30 ± 7 min was determined over the course of four experiments, one of which is displayed in Figure S37. While the half-life under these conditions does not mimic the biological environments the drugs would be exposed to, this study gives an indication of their stability when exposed to reducing agents and confirms that they do reduce to their platinum(II) congeners, which is essential for the mechanism and potent activity of these complexes.

### 3.5. Lipophilicity Studies

It is important to explore the lipophilicity of a drug, as it is widely believed that increased lipophilicity results in greater cellular accumulation [53]. To understand the lipophilicity of **1**–**4**, RP-HPLC was utilised to determine the $\log k_{w}$ of each complex. To calculate this, each complex was eluted at different isocratic percentages, whereby the retention time was used in Equation (1) to calculate the capacity factor (*k*). The percentage of the organic solvent was then plotted against log *k* for each isocratic percentage, producing a linear equation that allowed for $\log k_{w}$ to be deduced using Equation (2) (Figure S38). The $\log k_{w}$ value represents the capacity factor of the compound in 100% water and can be used to compare a series of compounds. Since **1**–**4** comprise the same axially bound ligand, biotin, the lipophilicity was influenced by the groups on the P_L_. The $\log k_{w}$ of each complex is presented in Table 1, with their corresponding P_L_. The increasing lipophilicity of the complexes followed the order: **1** < **4** < **2** < **3**. As expected, the increasing methyl groups on the P_L_ resulted in a higher $\log k_{w}$ value and the methoxy substituents had the greatest lipophilicity.

### 3.6. In Vitro Cytotoxicity

**Figure 8:**
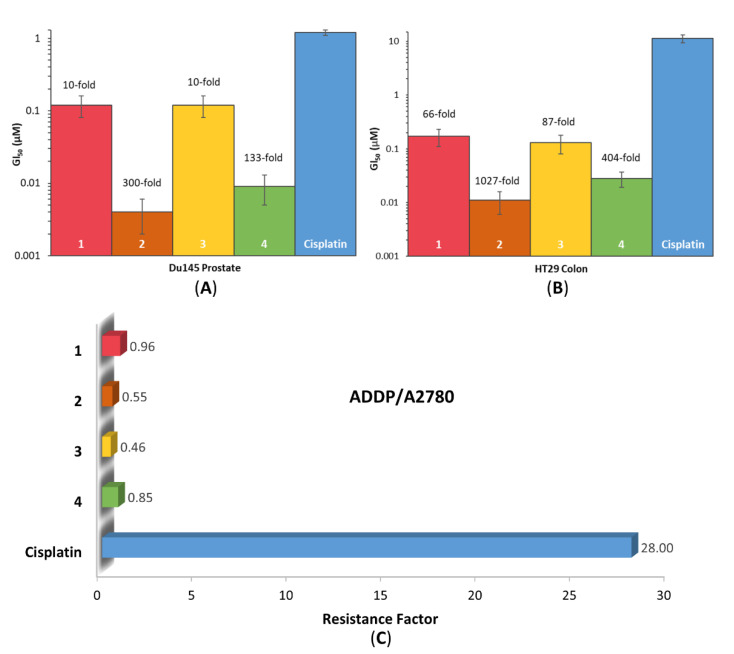
The GI_50_ values of **1**–**4** and cisplatin in the Du145 Prostate (**A**) and HT29 colon (**B**) cancer cell lines with the number-fold potency of **1**–**4** against cisplatin. (**C**) The resistance factors (RF) of **1**–**4** and cisplatin toward ADDP (subclone of A2780) versus A2780 ovarian cancer cell lines. Complexes were incubated for 72 h. GI_50_ values (µM) are the mean and reported with standard error of the mean; produced from three independent experiments.

The in vitro cytotoxicity of the platinum(II) and platinum(IV) precursors, biotin, and **1**–**4** was determined using MTT (3-(4,5-dimethylthiazol-2-yl)-2,5-diphenyltetrazolium bromide) assay against a range of cancer cell lines including HT29 colon, U87 glioblastoma, MCF-7 breast, A2780 ovarian, H460 lung, A431 skin, Du145 prostate, BE2-C neuroblastoma, SJ-G2 glioblastoma, MIA pancreas, and ADDP cisplatin-resistant ovarian, as well as the non-cancerous MCF10A breast cell line (Table S4). All complexes exhibited exceptional activity, with the most potent being **2**, with a GI_50_ of 4 nM in the Du145 prostate cancer cell line (Figure 8A). All complexes showed significantly better activity than cisplatin, carboplatin, and oxaliplatin in almost all cancer cell lines tested, with **2** exhibiting more than 1000-fold greater activity than cisplatin in the HT29 colon cancer cell line (Figure 8B).

Drug resistance is one of the major limitations of currently used platinum-based chemotherapeutics [13]. To assess the ability of **1**–**4** to overcome the acquired resistance of cisplatin, the resistance factor (RF) was calculated. The RF value is determined as a ratio of the GI_50_ values of a complex in a resistant and sensitive cell line. The RF of **1**–**4** and cisplatin were determined using the GI_50_ values in the cisplatin-resistant ADDP (subclone of A2780) and A2780 ovarian cancer cell lines (Figure 8C). The RF of cisplatin was calculated to be 28, whilst the RF of complexes **1**–**4** were all less than 1, with the order of complexes with decreasing RF being: cisplatin > **1** > **4** > **2** > **3**. This indicates that complexes **1**–**4** are able to overcome cisplatin resistance against the ADDP cell line.

The average GI_50_ value was calculated for each complex across all cancer cell lines. The order of increasing cytotoxicity is: **3** < **1** < **4** < **2**. These values were recorded in Table 1 with their corresponding $\log k_{w}$ values. Table 1 shows that the prodrug with the P_L_ 56Me_2_Phen (**2**) was the most cytotoxic (average GI_50_ = 0.027 µM), followed by that with the P_L_ 5MePhen (**4**, 0.055 µM), and lastly the prodrugs with the P_L_ Phen (**1**) and 47O_2_Me_2_Phen (**3**) exhibited average GI_50_ values that were comparable (0.455 µM and 0.459 µM, respectively). This follows the same order as the cytotoxicity of the platinum(II) cores, supporting that the prodrugs are reducing to their platinum(II) precursors within the cell [18,47,48]. There is a correlation between the cytotoxicity and RF of these complexes; as the cytotoxicity increases, the RF value decreases, indicating that the more potent the complex, the greater its ability to overcome cisplatin resistance. It is interesting to note, however, that **3** was an exception to this. It was the least potent complex out of **1**–**4**, yet had the lowest RF value (0.46), indicating it has the greatest ability to overcome cisplatin resistance. There is also a correlation between the lipophilicity and average GI_50_ values of **1**–**4**; however, **3**, again, was the exception. For **1**, **2**, and **4**, as the lipophilicity increased ($\log k_{w}$ values increased), the cytotoxicity also increased (GI_50_ values decreased), suggesting that increased lipophilicity may contribute to the cytotoxicity of these complexes.

**Figure 9:**
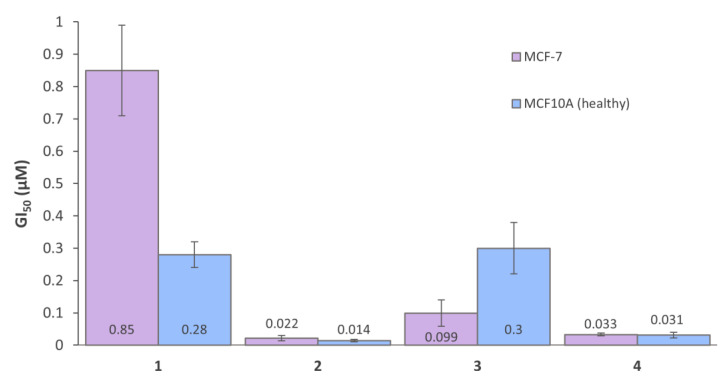
The GI_50_ values of **1**–**4** against MCF-7 (breast cancer) and MCF10A (healthy breast) cell lines. Complexes were incubated for 72 h. GI_50_ values (µM) are the mean and reported with standard error of the mean; produced from three independent experiments.

Whilst **1**–**4** each exhibited outstanding cytotoxicity in the cancerous cell lines, they were also active in the non-cancerous MCF10A breast cell line. However, **3**, comprising of the P_L_ 47O_2_Me_2_Phen, displayed more than 3-fold selectivity towards the cancerous breast (MCF-7) cell line, compared to the healthy breast (MCF10A) cell line (Figure 9). Biotin is overexpressed on numerous cancer types, particularly the MCF-7 breast cell line, which has a high degree of biotin expression, whilst the healthy breast (MCF10A) cell line has no biotin expression [45]. The platinum(II) and platinum(IV) precursors (**47OMESS(II)** and **47OMESS(IV)**) also exhibited selectivity towards this cancer cell line (3- and 7-fold, respectively), so it is not clear whether the selectivity exhibited by **3** is due to the platinum(II) core or the axial biotin moiety [49].

**Figure 10:**
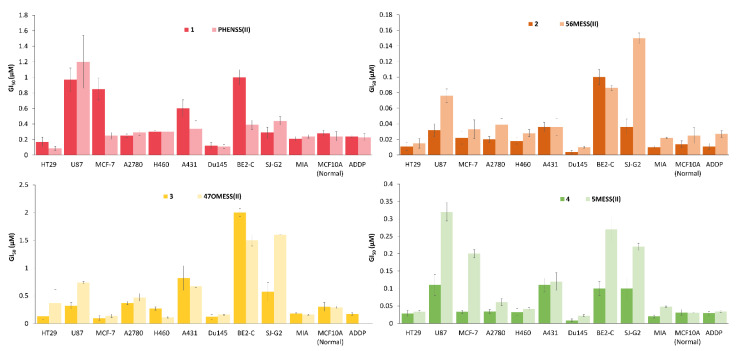
The GI_50_ values of **1**–**4** and their platinum(II) precursors, **PHENSS(II)**, **56MESS(II)**, **47OMESS(II)**, and **5MESS(II)**, in the cell lines tested against. The values are also listed in Table S4. Complexes were incubated for 72 h. GI_50_ values (µM) are the mean and reported with standard error of the mean; produced from three independent experiments.

To investigate the influence of the axially bound biotin ligand on the activity of each complex, the cytotoxicity of **1**–**4** were compared to their platinum(II) precursors (Figure 10). Of them, **1**, **3**, and **4** exhibited comparable toxicity in the non-cancerous MCF10A breast cell line compared to their platinum(II) precursors. Across all cancer cell lines, **1**, comprising of the PHENSS core, showed comparable activity to its platinum(II) precursor, whilst **2**, with the 56MESS core, exhibited ~2-fold enhanced activity compared to **56MESS(II)**. Despite **1** and **2** exhibiting low GI_50_ values (4–100 nM), they did not display any selectivity, particularly towards the MCF-7 cell line, which is overexpressed with the biotin receptor. Conversely, **3** and **4** both displayed interesting results. In all the cell lines tested, **3** exhibited the greatest activity against MCF-7 breast cancer cells, and compared to its **47OMESS(II)** precursor, **3** exhibited ~1.3-fold enhanced activity across the cancer cell lines. Additionally, **4** also exhibited increased activity (~2.3-fold) compared to its precursor, **5MESS(II)**, across all cancer cell lines, particularly in the MCF-7 breast cell line, where it was 6-fold more active. These results suggest that the biotin moiety may be improving the selectivity of both **3** and **4** in MCF-7 breast cancer cells. Whilst **3** was not necessarily the most potent complex from this series, it was, on average, still 8-fold more active than cisplatin against all cancer cells, and this, together with the selectivity **3** exhibited towards breast cancer cells compared to breast healthy cells, make it unique from this series of compounds.

### 3.7. Platinum Uptake Studies

**Figure 11:**
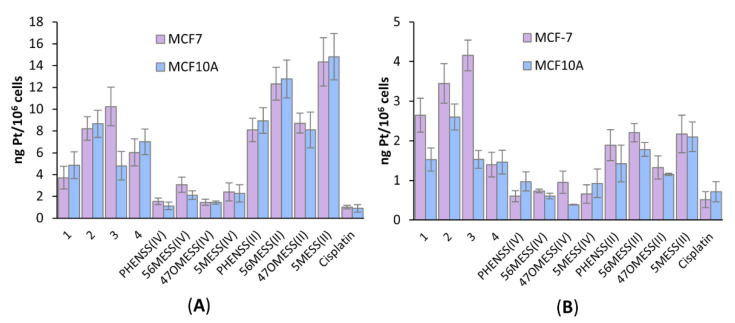
Cellular accumulation levels of **1**–**4**, their platinum(II) and platinum(IV) precursors and cisplatin against MCF-7 (breast cancer) and MCF10A (healthy breast) cells that were treated for 4 h, at 1.0 µM (**A**) and 0.1 µM (**B**) concentrations. Values are reported in ng/10^6^ cells and are reported with standard error of the mean; produced from three independent experiments.

Platinum uptake studies of **1**–**4**, their platinum(II) and platinum(IV) precursors, as well as cisplatin, were undertaken against the MCF-7 (breast cancer, high degree of biotin expression) and MCF10A (healthy breast, no biotin expression) cell lines. Cells were exposed to the complexes for 4 h, at 1.0 and 0.1 µM concentrations and ICP-MS was utilised to determine the cellular platinum uptake levels (Figure 11). At 1.0 µM, the platinum(II) complexes exhibited the greatest uptake, followed by prodrugs **1**–**4**, then the platinum(IV) di-hydroxido complexes, with cisplatin exhibiting the lowest uptake (Figure 11A). It is interesting to note that at the lower concentration (0.1 µM), prodrugs **1**–**3** exhibited the greatest uptake in the MCF-7 breast cancer cell line (2.6–4.2 ng/million cells), whereas their uptake in the MCF10A healthy cell line was ~1.8-fold lower and comparable to that of the platinum(II) precursors in both cell lines (Figure 11B). The platinum(II) precursors displayed only marginally greater uptake (1–1.3-fold, at 0.1 µM) in the MCF-7 cancer cell line compared to the MCF10A non-cancerous cell line. These results suggest that the biotin moiety may be contributing to the greater uptake of **1**–**3** in the MCF-7 breast cancer cell line at 0.1 µM. At both concentrations (1.0 and 0.1 µM), the uptake of **4** in the MCF-7 cell line was ~2-fold lower than its platinum(II) precursor, suggesting that the biotin moiety was not increasing its uptake at these concentrations. This is in contrast to its exhibited cytotoxicity, where **4** was shown to have 6-fold greater activity in MCF-7 breast cancer cells, compared to its platinum(II) precursor. At 0.1 µM, **3** exhibited the greatest uptake in MCF-7 breast cancer cells when compared to all the compounds, whilst also exhibiting almost 3-fold higher uptake towards the cancer (MCF-7) cell line over the non-cancerous (MCF10A) cell line (Figure 11B). This corresponds with the cytotoxicity exhibited by **3**, where it was shown to be 3-fold selective towards MCF-7 breast cancer cells compared to MCF10A non-cancerous cells.

When comparing the cellular uptake levels to resulting cytotoxicity, there was no clear trend in the MCF-7 cell line; however, a linear trend was observed in the MCF10A cell line when comparing the prodrugs to one another and when comparing the platinum(II) complexes to one another, but not necessarily when comparing the prodrugs to platinum(II) complexes (Figure S39). The discrepancy in correlation between cellular uptake and cytotoxicity might be due to the selected concentrations. At 1.0 µM, the platinum(II) complexes exhibited greater uptake, while at 0.1 µM, the prodrugs exhibited greater uptake (more so in the MCF-7 cell line), indicating that concentration does influence the resulting uptake levels of the different groups, relative to one another. In saying this, the trends in uptake levels between the different P_L_s appear to be consistent at both concentrations. The platinum(IV) di-hydroxido complexes exhibited consistently lower uptake at both concentrations. These results indicate that it is not the P_L_ that is being affected by the concentration, but rather the effect of the coordination sphere/conjugation of the biotin moiety. Whilst the 0.1 µM concentration seems more appropriate for these complexes (due to their high toxicity), it may be that additional concentrations are required to fully understand how the biotin moiety is influencing cellular accumulation levels. These results indicate that the biotin moiety is influencing cellular uptake, which in some cases is influencing the cytotoxicity of these complexes, but there are additional factors influencing their cytotoxicity.

**Figure 12:**
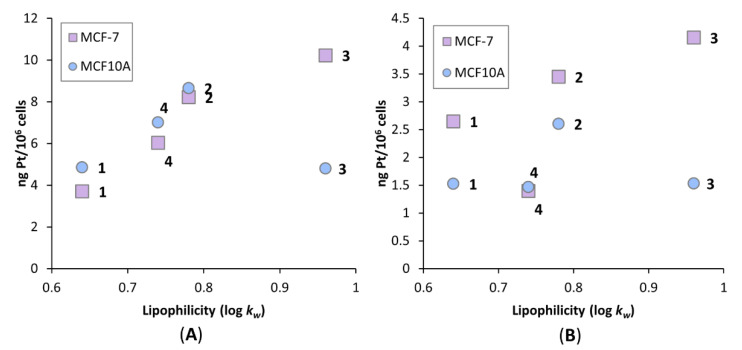
Correlation between the lipophilicity and cellular accumulation of **1**–**4** in MCF-7 (breast cancer) and MCF10A (healthy breast) cell lines. Lipophilicity values are reported as log *k_w_* values and cellular accumulation levels are reported as ng Pt/10^6^ cells, produced from three independent experiments. For cellular accumulation experiments, cells were treated for 4 h, at 1.0 µM (**A**) and 0.1 µM (**B**) concentrations.

To determine whether lipophilicity is influencing uptake, the cellular uptake levels of **1**–**4** were plotted against the log *k_w_* values obtained from the lipophilicity studies (Figure 12). The order of complexes in increasing lipophilicity are: **1** < **4** < **2** < **3**. There appears to be a correlation between increasing lipophilicity and increasing uptake levels of the complexes, more so in the MCF-7 cell line. In the MCF10A cell line, the common outlier present at both concentrations is **3**, which exhibited the lowest uptake levels of the four complexes in the healthy cell line. This could be attributed to the selectivity exhibited by **3** towards MCF-7, resulting in its lower uptake in the MCF10A cell line. Previous studies with similar complexes demonstrated no clear correlation between increasing lipophilicity and increasing cellular uptake; however, one of these studies utilised long chain fatty acids as the axial ligands, while the other utilised COX inhibitors, and neither of these previously tested drugs comprised of the 47OMESS core [24,48]. Rather than comparing the lipophilicity of different axial ligands to one another, here, we have compared prodrugs comprised of the same axial ligand conjugated to different platinum cores. This indicates that increasing the lipophilicity of the platinum core results in the greater cellular uptake. It is apparent that lipophilicity of the platinum core and selectivity from the biotin moiety are both contributing factors to the increased cellular uptake of **1**–**4**. In some cases, this translated to the resulting cytotoxicity; however, it was dependent on concentration and the choice of cell line. The discrepancy in correlation between cellular uptake and cytotoxicity could be due to the mechanisms by which these prodrugs cause cell death.

## 4. Conclusions

A series of platinum(IV) prodrugs of the type [Pt(P_L_)(A_L_)(biotin)(OH)]^2+^ (where P_L_ = Phen or a substituted variant of Phen and A_L_ = SSDACH) comprised of a biotin moiety to selectively target cancer cells were synthesised and characterised before their biological activity was assessed. The structure and purity of the complexes were confirmed by NMR spectroscopy, HPLC, ESI-MS, UV, and CD spectroscopy. Reduction studies for **3** were undertaken using ^1^H NMR spectroscopy and confirmed the release of the axially bound biotin ligand, with a half-life of ~30 min. RP-HPLC was employed to assess the lipophilicity of the complexes, producing log *k_w_* values, which determined the order of increasing lipophilicity to be: **1** < **4** < **2** < **3**.

The in vitro cytotoxicity of the complexes was assessed using MTT assays and showed that **2** was the most cytotoxic in this series, exhibiting a GI_50_ of 4 nM in the Du145 prostate cancer cell line. All complexes displayed significantly enhanced cytotoxicity compared to cisplatin against all the cell lines tested, with **2** being more than 1000-fold more active in the HT29 colon cancer cell line relative to cisplatin. The addition of the biotin moiety to the axial position proved to enhance the activity of **2**, **3**, and **4** in the MCF-7 (breast, high biotin expression) cancer cell line when compared to their platinum(II) precursors. Prodrug **3** and its precursors (**47OMESS(II)** and **47OMESS(IV)**) also exhibited 3–7-fold selectivity towards MCF-7 breast cancer cells compared to MCF10A breast healthy cells, indicating this selectivity may be a combination of the 47OMESS core and the axially bound biotin ligand.

To further elucidate the activity of the complexes, ICP-MS was used to determine platinum cellular uptake levels in MCF-7 and MCF10A cells. At 0.1 µM, **3** exhibited the greatest uptake in the MCF-7 cell line, almost 3 times greater than the MCF10A cell line, which is comparable to the trend observed for its cytotoxicity in these cell lines. There appeared to be a slight correlation between increasing cellular uptake and increasing cytotoxicity of the compounds in the MCF10A cell line, but only when comparing the prodrugs to one another, and the platinum(II) complexes to one another. At 0.1 µM, the biotin moiety was shown to improve the cellular uptake of **1**–**3** in the MCF-7 cell line (by ~1.8-fold) when compared to their platinum(II) precursors and compared to their uptake levels in the MCF10A cell line. It is apparent that the selectivity from the biotin moiety is influencing the cellular accumulation levels, but additional drug concentrations are required to understand how this is influencing their resulting cytotoxicity. There was an increasing linear trend observed with the lipophilicity and cellular uptake of the complexes, suggesting that lipophilicity of the platinum core may also be a contributing factor to the activity of these complexes. These findings suggest that the selectivity from the biotin moiety and lipophilicity of the platinum core are contributing factors to the cellular uptake of **1**–**4**; however, this was not always translated to the observed cytotoxicity, indicating that additional factors are contributing to their activity.

## 5. Patents

This work is part of an Australian Provisional Patent Application 2022900110, Platinum(IV) complexes, February 2022, Western Sydney University, Australia.

## Figures and Tables

**Table 1:** The $\log k_{w}$ and average GI_50_ of each complex, with its corresponding P_L_. Average GI_50_ values were calculated from the panel of cancer cell lines tested (Table S4).

Complex	$\log k_{w}$	P_L_	Average GI_50_ (µM)
**1**	0.64	Phen	0.455
**4**	0.74	5MePhen	0.055
**2**	0.78	56Me_2_Phen	0.027
**3**	0.96	47O_2_Me_2_Phen	0.459

## Data Availability

All data relevant to the publication are included.

## Supplementary Materials

The following supporting information can be downloaded at: https://www.mdpi.com/article/10.3390/pharmaceutics14122780/s1, Table S1: The flash chromatography conditions for **1**–**4**, including flow rates, gradients, and elution of complexes represented by column volume (CV). Table S2: The number of cells per well for each cell line and replicate. Figure S1: The ^1^H NMR spectrum of biotin-NHS ester in DMSO-d_6_ at 298 K. Insert: The chemical structure of biotin-NHS ester with assigned numbering. Figure S2: The COSY NMR spectrum of biotin-NHS ester in DMSO-d_6_ at 298 K. Figure S3: The ^1^H NMR spectrum of [Pt(Phen)(SSDACH)(Biotin)(OH)](NO_3_)_2_ (**1**) in D_2_O at 298 K. Insert: The chemical structure of **1** with assigned numbering. Figure S4: The COSY NMR spectrum of [Pt(Phen)(SSDACH)(Biotin)(OH)](NO_3_)_2_ (**1**) in D_2_O at 298 K. Figure S5: The ^195^Pt NMR spectrum of [Pt(Phen)(SSDACH)(Biotin)(OH)](NO_3_)_2_ (**1**) in D_2_O at 298 K, showing a peak at 539 ppm. Figure S6: The ^1^H-^195^Pt HMQC NMR spectrum of [Pt(Phen)(SSDACH)(Biotin)(OH)](NO_3_)_2_ (**1**) in D_2_O at 298 K. Figure S7: The ^1^H NMR spectrum of [Pt(56Me_2_Phen)(SSDACH)(Biotin)(OH)](NO_3_)_2_ (**2**) in D_2_O at 298 K. Insert: The chemical structure of **2** with assigned numbering. Figure S8: The COSY NMR spectrum of [Pt(56Me_2_Phen)(SSDACH)(Biotin)(OH)](NO_3_)_2_ (**2**) in D_2_O at 298 K. Figure S9: The ^195^Pt NMR spectrum of [Pt(56Me_2_Phen)(SSDACH)(Biotin)(OH)](NO_3_)_2_ (**2**) in D_2_O at 298 K, showing a peak at 526 ppm. Figure S10: The ^1^H-^195^Pt HMQC NMR spectrum of [Pt(56Me_2_Phen)(SSDACH)(Biotin)(OH)](NO_3_)_2_ (**2**) in D_2_O at 298 K. Figure S11: The ^1^H NMR spectrum of [Pt(47O_2_Me_2_Phen)(SSDACH)(Biotin)(OH)](NO_3_)_2_ (**3**) in D_2_O at 298 K. Insert: The chemical structure of **3** with assigned numbering. Figure S12: The COSY NMR spectrum of [Pt(47O_2_Me_2_Phen)(SSDACH)(Biotin)(OH)](NO_3_)_2_ (**3**) in D_2_O at 298 K. Figure S13: The ^195^Pt NMR spectrum of [Pt(47O_2_Me_2_Phen)(SSDACH)(Biotin)(OH)](NO_3_)_2_ (**3**) in D_2_O at 298 K, showing a peak at 590 ppm. Figure S14: The ^1^H-^195^Pt HMQC NMR spectrum of [Pt(47O_2_Me_2_Phen)(SSDACH)(Biotin)(OH)](NO_3_)_2_ (**3**) in D_2_O at 298 K. Figure S15: The ^1^H NMR spectrum of [Pt(5MePhen)(SSDACH)(Biotin)(OH)](NO_3_)_2_ (**4**) in D_2_O at 298 K. Insert: The chemical structure of **4** with assigned numbering. Figure S16: The COSY NMR spectrum of [Pt(5MePhen)(SSDACH)(Biotin)(OH)](NO_3_)_2_ (**4**) in D_2_O at 298 K. Figure S17: The ^195^Pt NMR spectrum of [Pt(5MePhen)(SSDACH)(Biotin)(OH)](NO_3_)_2_ (**4**) in D_2_O at 298 K, showing a peak at 540 ppm. Figure S18: The ^1^H-^195^Pt HMQC NMR spectrum of [Pt(5MePhen)(SSDACH)(Biotin)(OH)](NO_3_)_2_ (**4**) in D_2_O at 298 K. Figure S19: The HPLC chromatogram of [Pt(Phen)(SSDACH)(Biotin)(OH)](NO_3_)_2_ (**1**), at 254 nm, at a gradient of 0–100% (ACN:H_2_O, 9:1) over 15 min. Figure S20: The HPLC chromatogram of [Pt(56Me_2_Phen)(SSDACH)(Biotin)(OH)](NO_3_)_2_ (**2**), at 254 nm, at a gradient of 0–100% (ACN:H_2_O, 9:1) over 15 min. Figure S21: The HPLC chromatogram of [Pt(47O_2_Me_2_Phen)(SSDACH)(Biotin)(OH)](NO_3_)_2_ (**3**), at 254 nm, at a gradient of 0–100% (ACN:H_2_O, 9:1) over 15 min. Figure S22: The HPLC chromatogram of [Pt(5MePhen)(SSDACH)(Biotin)(OH)](NO_3_)_2_ (**4**), at 254 nm, at a gradient of 0–100% (ACN:H_2_O, 9:1) over 15 min. Figure S23: Full ESI-MS spectrum of [Pt(Phen)(SSDACH)(Biotin)(OH)](NO_3_)_2_ (**1**). Below: The expanded region of the [M-H]^+^ peak. Figure S24: Full ESI-MS spectrum of [Pt(56Me_2_Phen)(SSDACH)(Biotin)(OH)](NO_3_)_2_ (**2**). Below: The expanded region of the [M-H]^+^ peak. Figure S25: Full ESI-MS spectrum of [Pt(47O_2_Me_2_Phen)(SSDACH)(Biotin)(OH)](NO_3_)_2_ (**3**). Below: The expanded region of the [M-H]^+^ peak. Figure S26: Full ESI-MS spectrum of [Pt(5MePhen)(SSDACH)(Biotin)(OH)](NO_3_)_2_ (**4**). Below: The expanded region of the [M-H]^+^ peak. Table S3: Summary of characterisation data for **1**–**4**. Figure S27: The UV spectrum of a replicate of [Pt(Phen)(SSDACH)(Biotin)(OH)](NO_3_)_2_ (**1**) in water. Figure S28: The UV spectrum of a replicate of [Pt(56Me_2_Phen)(SSDACH)(Biotin)(OH)](NO_3_)_2_ (**2**) in water. Figure S29: The UV spectrum of a replicate of [Pt(47O_2_Me_2_Phen)(SSDACH)(Biotin)(OH)](NO_3_)_2_ (**3**) in water. Figure S30: The UV spectrum of a replicate of [Pt(5MePhen)(SSDACH)(Biotin)(OH)](NO_3_)_2_ (**4**) in water. Figure S31: The CD spectrum of [Pt(Phen)(SSDACH)(Biotin)(OH)](NO_3_)_2_ (**1**) in water. 9pt smoothing applied. Figure S32: The CD spectrum of [Pt(56Me_2_Phen)(SSDACH)(Biotin)(OH)](NO_3_)_2_ (**2**) in water. 9pt smoothing applied. Figure S33: The CD spectrum of [Pt(47O_2_Me_2_Phen)(SSDACH)(Biotin)(OH)](NO_3_)_2_ (**3**) in water. 9pt smoothing applied. Figure S34: The CD spectrum of [Pt(5MePhen)(SSDACH)(Biotin)(OH)](NO_3_)_2_ (**4**) in water. 9pt smoothing applied. Figure S35: The Pt(IV) region in the ^195^Pt NMR spectrum of **3** after reduction, showing the disappearance of the peak at 590 ppm. Figure S36: The Pt(II) region in the ^195^Pt NMR spectrum of **3** after reduction, showing a new peak at −2801 ppm. Figure S37: A replicate of the time course reduction study of **3** via ^1^H NMR. The Pt(II) and Pt(IV) resonances are plotted as a percentage. Figure S38: Plot of the concentration of organic solvent vs. $\log k_{w}$ for **1**–**4**. Table S4: The in vitro cytotoxicity values of complexes **1**–**4**, their platinum(II) and platinum(IV) precursors, biotin, cisplatin, carboplatin and oxaliplatin. GI_50_ values (µM) are reported with standard error of the mean; produced from experiments that were conducted on three separate occasions (n = 3); n.d. = not determined. Resistance factor (RF) defined as GI_50_ in ADDP/GI_50_ in A2780. Figure S39: The correlation between the cellular accumulation and cytotoxicity of **1**–**4** and their platinum(II) precursors against MCF-7 (breast cancer, (**A**) and (**C**)) and MCF10A (normal breast, (**B**) and (**D**)) cells that were treated for 4 h, at 1.0 µM ((**A**) and (**B**)) and 0.1 µM ((**C**) and (**D**)) concentrations.

## References

[1] Sung H., Ferlay J., Siegel R.L., Laversanne M., Soerjomataram I., Jemal A., Bray F. (2021). Global cancer statistics 2020: GLOBOCAN estimates of incidence and mortality worldwide for 36 cancers in 185 countries. CA A Cancer J. Clin..

[2] Johnstone T.C., Park G.Y., Lippard S.J. (2014). Understanding and improving platinum anticancer drugs–phenanthriplatin. Anticancer Res..

[3] Rosenberg B., Van Camp L., Krigas T. (1965). Inhibition of Cell Division in Escherichia coli by Electrolysis Products from a Platinum Electrode. Nature.

[4] Rosenberg B., Van Camp L., Trosko J.E., Mansour V.H. (1969). Platinum compounds: A new class of potent antitumour agents. Nature.

[5] Rottenberg S., Disler C., Perego P. (2021). The rediscovery of platinum-based cancer therapy. Nat. Rev. Cancer.

[6] Jamieson E.R., Lippard S.J. (1999). Structure, recognition, and processing of cisplatin− DNA adducts. Chem. Rev..

[7] Bruno P.M., Liu Y., Park G.Y., Murai J., Koch C.E., Eisen T.J., Pritchard J.R., Pommier Y., Lippard S.J., Hemann M.T. (2017). A subset of platinum-containing chemotherapeutic agents kills cells by inducing ribosome biogenesis stress. Nat. Med..

[8] Deo K.M., Ang D.L., McGhie B., Rajamanickam A., Dhiman A., Khoury A., Holland J., Bjelosevic A., Pages B., Gordon C. (2018). Platinum coordination compounds with potent anticancer activity. Coord. Chem. Rev..

[9] Khoury A., Deo K.M., Aldrich-Wright J.R. (2020). Recent advances in platinum-based chemotherapeutics that exhibit inhibitory and targeted mechanisms of action. J. Inorg. Biochem..

[10] Ivanova S. (2022). Comparative assessment of clinical trials, indications, pharmacokinetic parameters and side effects of approved platinum drugs. Pharmacia.

[11] Boulikas T., Pantos A., Bellis E., Christofis P. (2007). Designing platinum compounds in cancer: Structures and mechanisms. Cancer Ther..

[12] Wang X., Guo Z. (2013). Targeting and delivery of platinum-based anticancer drugs. Chem. Soc. Rev..

[13] Johnstone T.C., Suntharalingam K., Lippard S.J. (2016). The next generation of platinum drugs: Targeted Pt(II) agents, nanoparticle delivery, and Pt(IV) prodrugs. Chem. Rev..

[14] Wheate N.J., Taleb R.I., Krause-Heuer A.M., Cook R.L., Wang S., Higgins V.J., Aldrich-Wright J.R. (2007). Novel platinum(II)-based anticancer complexes and molecular hosts as their drug delivery vehicles. Dalton Trans..

[15] Brodie C.R., Collins J.G., Aldrich-Wright J.R. (2004). DNA binding and biological activity of some platinum(II) intercalating compounds containing methyl-substituted 1, 10-phenanthrolines. Dalton Trans..

[16] Kostrhunova H., Zajac J., Novohradsky V., Kasparkova J., Malina J., Aldrich-Wright J.R., Petruzzella E., Sirota R., Gibson D., Brabec V. (2019). A subset of new platinum antitumor agents kills cells by a multimodal mechanism of action also involving changes in the organization of the microtubule cytoskeleton. J. Med. Chem..

[17] Moretto J., Chauffert B., Ghiringhelli F., Aldrich-Wright J.R., Bouyer F. (2011). Discrepancy between in vitro and in vivo antitumor effect of a new platinum(II) metallointercalator. Investig. New Drugs.

[18] Macias F.J., Deo K.M., Wormell P., Clegg J.K., Zhang Y., Li F., Zheng G., Sakoff J., Gilbert J., Aldrich-Wright J.R. (2015). Synthesis and analysis of the structure, diffusion and cytotoxicity of heterocyclic platinum(IV) complexes. Chem. A Eur. J..

[19] Harper B.W., Petruzzella E., Sirota R., Faccioli F.F., Aldrich-Wright J.R., Gandin V., Gibson D. (2017). Synthesis, characterization and in vitro and in vivo anticancer activity of Pt(IV) derivatives of [Pt (1 S, 2 S-DACH)(5, 6-dimethyl-1, 10-phenanthroline)]. Dalton Trans..

[20] Hall M.D., Mellor H.R., Callaghan R., Hambley T.W. (2007). Basis for design and development of platinum(IV) anticancer complexes. J. Med. Chem..

[21] Hall M.D., Hambley T.W. (2002). Platinum(IV) antitumour compounds: Their bioinorganic chemistry. Coord. Chem. Rev..

[22] Gibson D. (2016). Platinum(IV) anticancer prodrugs–hypotheses and facts. Dalton Trans..

[23] Chen C.K., Zhang J.Z., Aitken J.B., Hambley T.W. (2013). Influence of equatorial and axial carboxylato ligands on the kinetic inertness of platinum(IV) complexes in the presence of ascorbate and cysteine and within DLD-1 cancer cells. J. Med. Chem..

[24] Deo K.M., Sakoff J., Gilbert J., Zhang Y., Wright J.R.A. (2019). Synthesis, characterisation and influence of lipophilicity on cellular accumulation and cytotoxicity of unconventional platinum (IV) prodrugs as potent anticancer agents. Dalton Trans..

[25] Bruijnincx P.C., Sadler P.J. (2008). New trends for metal complexes with anticancer activity. Curr. Opin. Chem. Biol..

[26] Johnstone T.C., Wilson J.J., Lippard S.J. (2013). Monofunctional and higher-valent platinum anticancer agents. Inorg. Chem..

[27] Ellis L.T., Er H.M., Hambley T.W. (1995). The influence of the axial ligands of a series of platinum(IV) anti-cancer complexes on their reduction to platinum(II) and reaction with DNA. Aust. J. Chem..

[28] Weaver E.L., Bose R.N. (2003). Platinum(II) catalysis and radical intervention in reductions of platinum(IV) antitumor drugs by ascorbic acid. J. Inorg. Biochem..

[29] Choi S., Filotto C., Bisanzo M., Delaney S., Lagasee D., Whitworth J.L., Jusko A., Li C., Wood N.A., Willingham J. (1998). Reduction and anticancer activity of platinum(IV) complexes. Inorg. Chem..

[30] Czapla-Masztafiak J., Kubas A., Kayser Y., Fernandes D.L.A., Kwiatek W.M., Lipiec E., Deacon G.B., Al-Jorani K., Wood B.R., Szlachetko J. (2018). Mechanism of hydrolysis of a platinum(IV) complex discovered by atomic telemetry. J. Inorg. Biochem..

[31] Galanski M., Jakupec M.A., Keppler B.K. (2005). Update of the preclinical situation of anticancer platinum complexes: Novel design strategies and innovative analytical approaches. Curr. Med. Chem..

[32] Rahman A., Roh J.K., Wolpert-DeFilippes M.K., Goldin A., Venditti J.M., Woolley P.V. (1988). Therapeutic and pharmacological studies of tetrachloro (d, l-trans) 1, 2-diaminocyclohexane platinum (IV)(tetraplatin), a new platinum analogue. Cancer Res..

[33] Chaney S.G., Wyrick S., Till G.K. (1990). In vitro biotransformations of tetrachloro (d, l-trans)-1, 2-diaminocyclohexaneplatinum (IV)(tetraplatin) in rat plasma. Cancer Res..

[34] Wheate N.J., Walker S., Craig G.E., Oun R. (2010). The status of platinum anticancer drugs in the clinic and in clinical trials. Dalton Trans..

[35] Sternberg C.N., Petrylak D.P., Sartor O., Witjes J.A., Demkow T., Ferrero J.-M., Eymard J.-C., Falcon S., Calabrò F., James N. (2009). Multinational, double-blind, phase III study of prednisone and either satraplatin or placebo in patients with castrate-refractory prostate cancer progressing after prior chemotherapy: The SPARC trial. J. Clin. Oncol..

[36] Theiner S., Varbanov H.P., Galanski M., Egger A.E., Berger W., Heffeter P., Keppler B.K. (2015). Comparative in vitro and in vivo pharmacological investigation of platinum(IV) complexes as novel anticancer drug candidates for oral application. JBIC J. Biol. Inorg. Chem..

[37] Ludwig J.A., Weinstein J.N. (2005). Biomarkers in cancer staging, prognosis and treatment selection. Nat. Rev. Cancer.

[38] Saha S., Majumdar R., Hussain A., Dighe R.R., Chakravarty A.R. (2013). Biotin-conjugated tumour-targeting photocytotoxic iron(III) complexes. Philos. Trans. R. Soc. A Math. Phys. Eng. Sci..

[39] Russell-Jones G., McTavish K., McEwan J., Rice J., Nowotnik D. (2004). Vitamin-mediated targeting as a potential mechanism to increase drug uptake by tumours. J. Inorg. Biochem..

[40] Chen S., Zhao X., Chen J., Chen J., Kuznetsova L., Wong S.S., Ojima I. (2010). Mechanism-based tumor-targeting drug delivery system. Validation of efficient vitamin receptor-mediated endocytosis and drug release. Bioconjugate Chem..

[41] Tripodo G., Mandracchia D., Collina S., Rui M., Rossi D. (2014). New perspectives in cancer therapy: The biotin-antitumor molecule conjugates. Med. Chem..

[42] Muhammad N., Sadia N., Zhu C., Luo C., Guo Z., Wang X. (2017). Biotin-tagged platinum(IV) complexes as targeted cytostatic agents against breast cancer cells. Chem. Commun..

[43] Mitra K., Shettar A., Kondaiah P., Chakravarty A.R. (2016). Biotinylated platinum(II) ferrocenylterpyridine complexes for targeted photoinduced cytotoxicity. Inorg. Chem..

[44] Zhao J., Hua W., Xu G., Gou S. (2017). Biotinylated platinum(IV) complexes designed to target cancer cells. J. Inorg. Biochem..

[45] Ren W.X., Han J., Uhm S., Jang Y.J., Kang C., Kim J.-H., Kim J.S. (2015). Recent development of biotin conjugation in biological imaging, sensing, and target delivery. Chem. Commun..

[46] Deo K.M., Sakoff J., Gilbert J., Zhang Y., Wright J.R.A. (2019). Synthesis, characterisation and potent cytotoxicity of unconventional platinum (IV) complexes with modified lipophilicity. Dalton Trans..

[47] Tarleton M., Gilbert J., Robertson M.J., McCluskey A., Sakoff J.A. (2011). Library synthesis and cytotoxicity of a family of 2-phenylacrylonitriles and discovery of an estrogen dependent breast cancer lead compound. Med. Chem. Commun..

[48] Khoury A., Sakoff J.A., Gilbert J., Scott K.F., Karan S., Gordon C.P., Aldrich-Wright J.R. (2022). Cyclooxygenase-inhibiting platinum(IV) prodrugs with potent anticancer activity. Pharmaceutics.

[49] Khoury A., Elias E., Mehanna S., Shebaby W., Deo K.M., Mansour N., Khalil C., Sayyed K., Sakoff J.A., Gilbert J. (2022). Novel Platinum(II) and Platinum(IV) Antitumor Agents that Exhibit Potent Cytotoxicity and Selectivity. J. Med. Chem..

[50] Aputen A.D., Elias M.G., Gilbert J., Sakoff J.A., Gordon C.P., Scott K.F., Aldrich-Wright J.R. (2022). Potent Chlorambucil-Platinum (IV) Prodrugs. Int. J. Mol. Sci..

[51] Garbutcheon-Singh K.B., Leverett P., Myers S., Aldrich-Wright J.R. (2013). Cytotoxic platinum(II) intercalators that incorporate 1 R, 2 R-diaminocyclopentane. Dalton Trans..

[52] Garbutcheon-Singh K.B., Aldrich-Wright J.R. (2017). Platinum intercalators of DNA as anticancer agents. Eur. J. Inorg. Chem..

[53] Oldfield S.P., Hall M.D., Platts J.A. (2007). Calculation of lipophilicity of a large, diverse dataset of anticancer platinum complexes and the relation to cellular uptake. J. Med. Chem..

