# Supplementary material for: Potent Platinum(IV) Prodrugs That Incorporate a Biotin Moiety to Selectively Target Cancer Cells
Source: Pharmaceutics. 2022 Dec 12;14(12):2780. doi: 10.3390/pharmaceutics14122780 (PMC9853328; doi:10.3390/pharmaceutics14122780)
Supplement: Supplementary file 1 [file pharmaceutics-14-02780-s001.zip › pharmaceutics-1967413 - supplementary new.pdf]

Supplementary Materials: Potent Platinum(IV) Prodrugs that Incorporate a Biotin Moiety to Selectively Target Cancer Cells

Aleen Khoury, Jennette A. Sakoff, Jayne Gilbert, Shawan Karan, Christopher P. Gordon and Janice R. Aldrich-Wright

1. Flash Chromatography

Table S1. The flash chromatography conditions for **1**–**4**, including flow rates, gradients and elution of complexes represented by column volume (CV).

Complex	Flowrate (mL/min)	Gradient (% MeOH (B)) over various CVs	Elution of complex (CV)
[Pt(Phen)(SSDACH)(Biotin)(OH)](NO$_3$)$_2$ (**1**)	4	0% B for 4 CV<br>0–20% B for 4 CV	5–7
[Pt(56Me$_2$Phen)(SSDACH)(Biotin)](OH) (NO$_3$)$_2$ (**2**)	4	0% B for 4 CV<br>0–30% B for 6 CV	7–9
[Pt(47O$_2$Me$_2$Phen)(SSDACH)(Biotin) (OH)](NO$_3$)$_2$ (**3**)	4	0% B for 5 CV<br>0–30% B for 7 CV	9–11
[Pt(5MePhen)(SSDACH)(Biotin)(OH)] (NO$_3$)$_2$ (**4**)	4	0% B for 4 CV<br>0–25% B for 5 CV	6–8

2. Cellular Accumulation

Table S2. The number of cells per well for each cell line and replicate.

Replicate	Cell line	Number of cells per well
1	MCF-7	2.20×10^5
	MCF10A	3.15×10^5
2	MCF-7	2.30×10^5
	MCF10A	1.10×10^5
3	MCF-7	3.20×10^5
	MCF10A	2.95×10^5

3. NMR Spectra

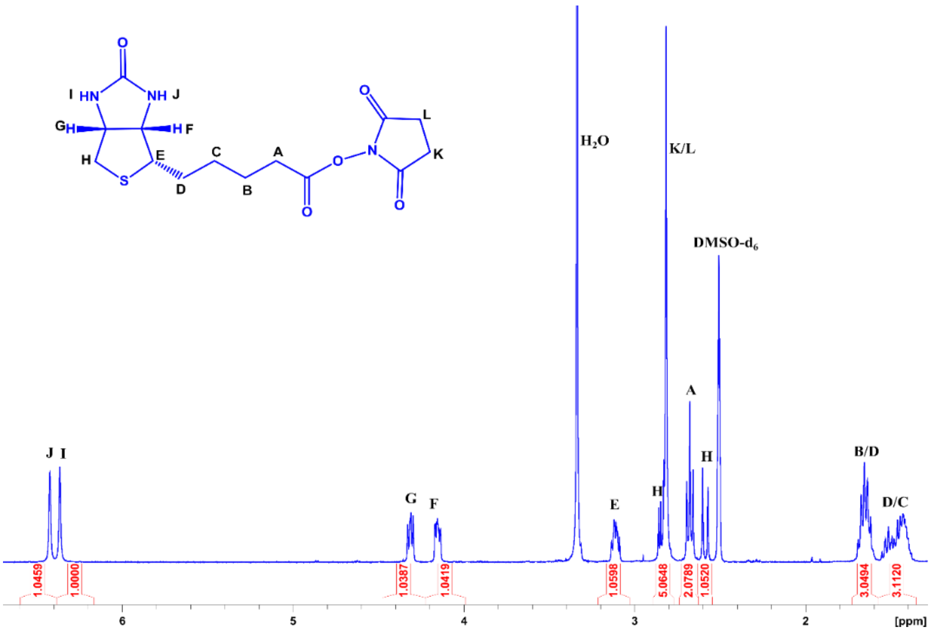

Figure S1. The ^{1}H NMR spectrum of biotin-NHS ester in DMSO-d$_6$ at 298 K. Insert: The chemical structure of biotin-NHS ester with assigned numbering.

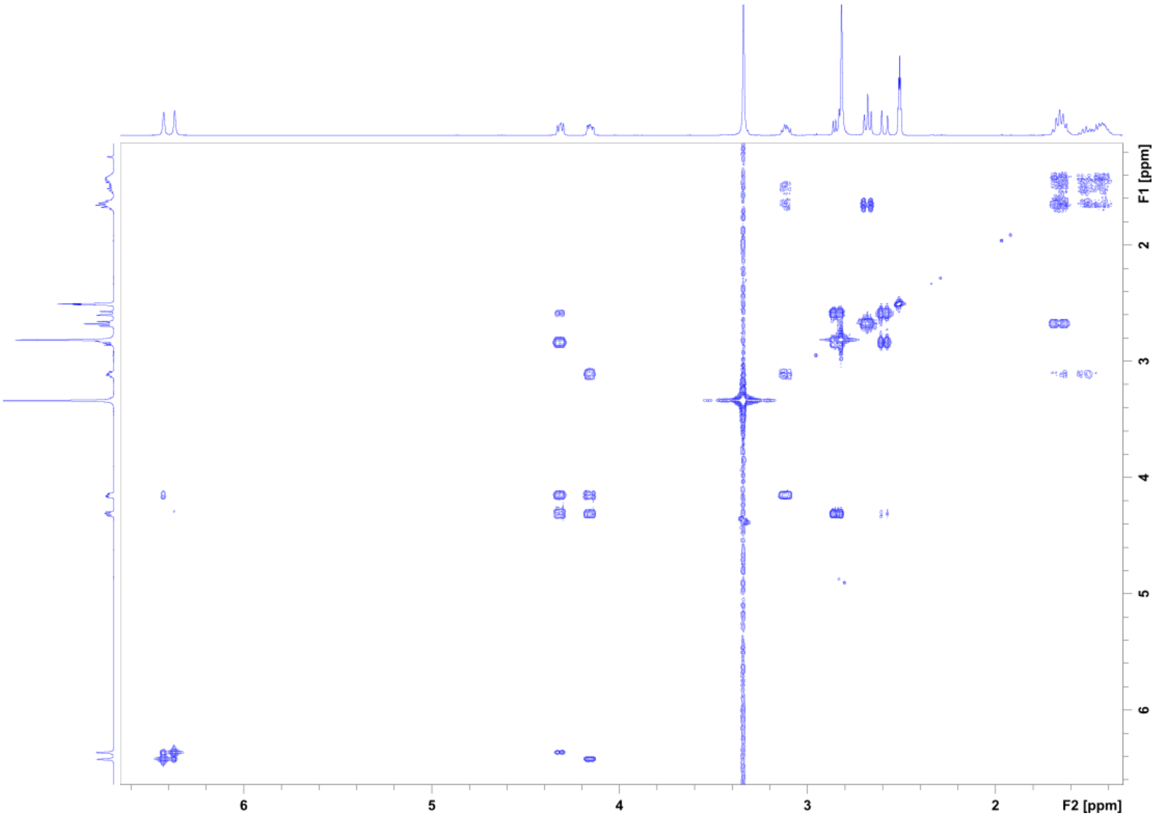

Figure S2. The COSY NMR spectrum of biotin-NHS ester in DMSO-d_6 at 298 K.

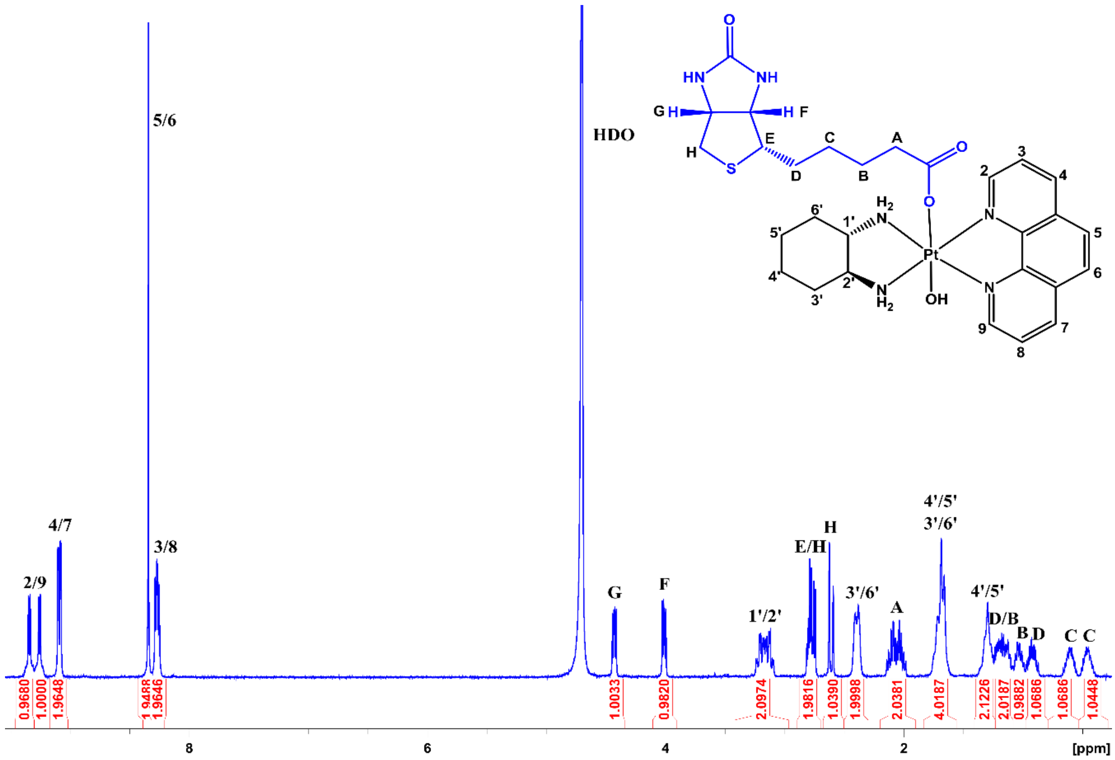

Figure S3. The ^{1}H NMR spectrum of $[Pt(Phen)(SSDACH)(Biotin)(OH)](NO_3)_2$ (**1**) in D_2O at 298 K. Insert: The chemical structure of **1** with assigned numbering.

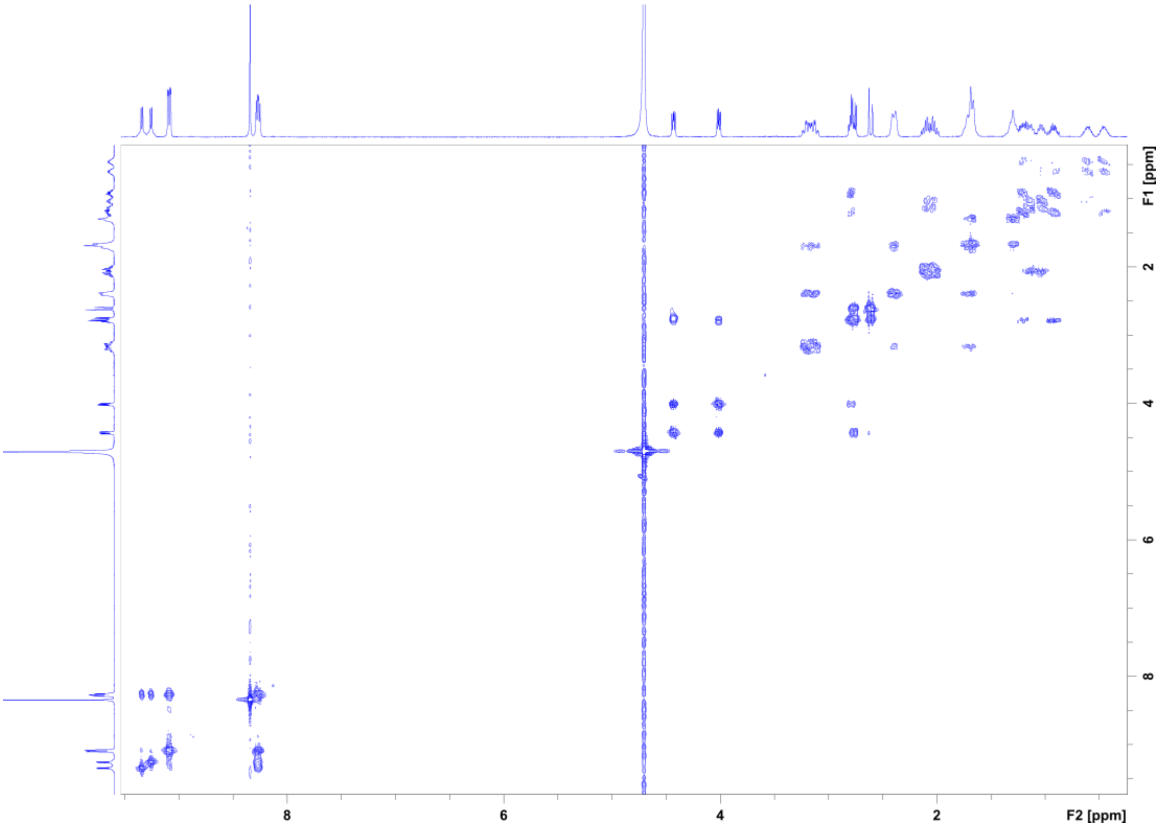

Figure S4. The COSY NMR spectrum of [Pt(Phen)(SSDACH)(Biotin)(OH)]$(NO_3)_2$ (**1**) in D_2O at 298 K.

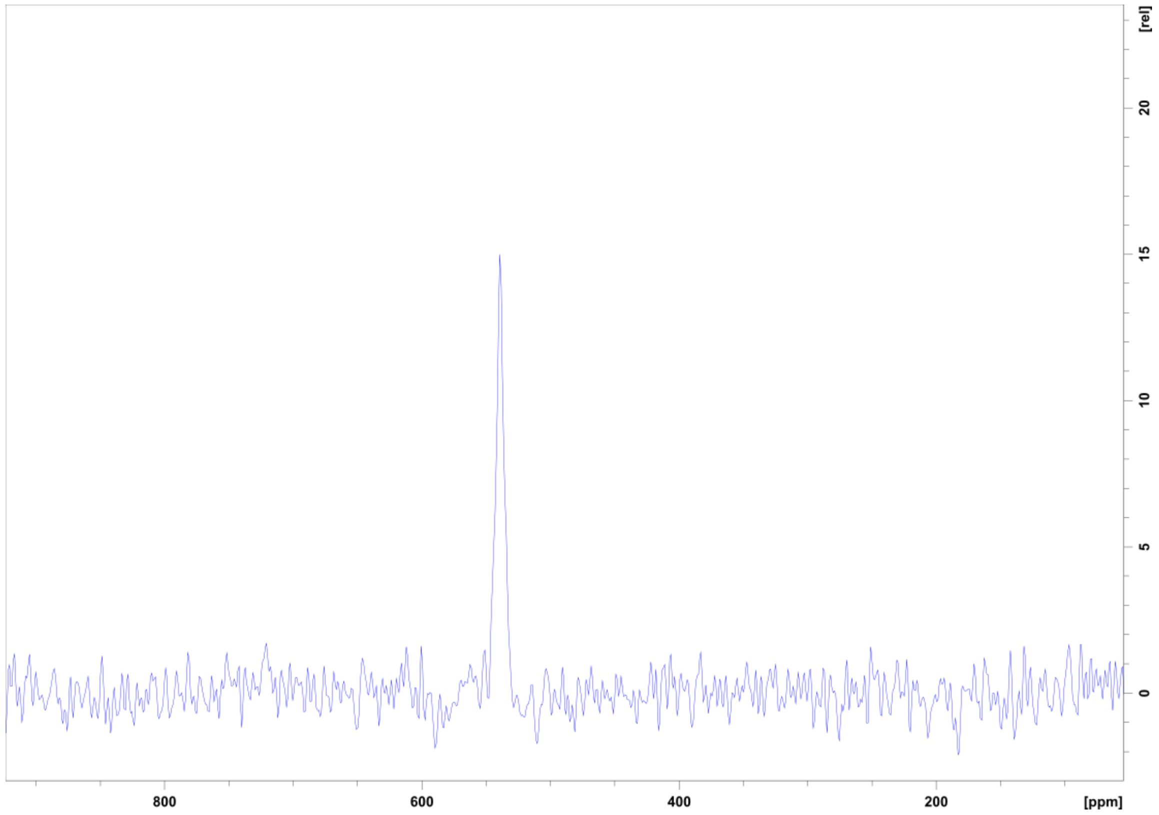

Figure S5. The ^{195}Pt NMR spectrum of [Pt(Phen)(SSDACH)(Biotin)(OH)]$(NO_3)_2$ (**1**) in D_2O at 298 K, showing a peak at 539 ppm.

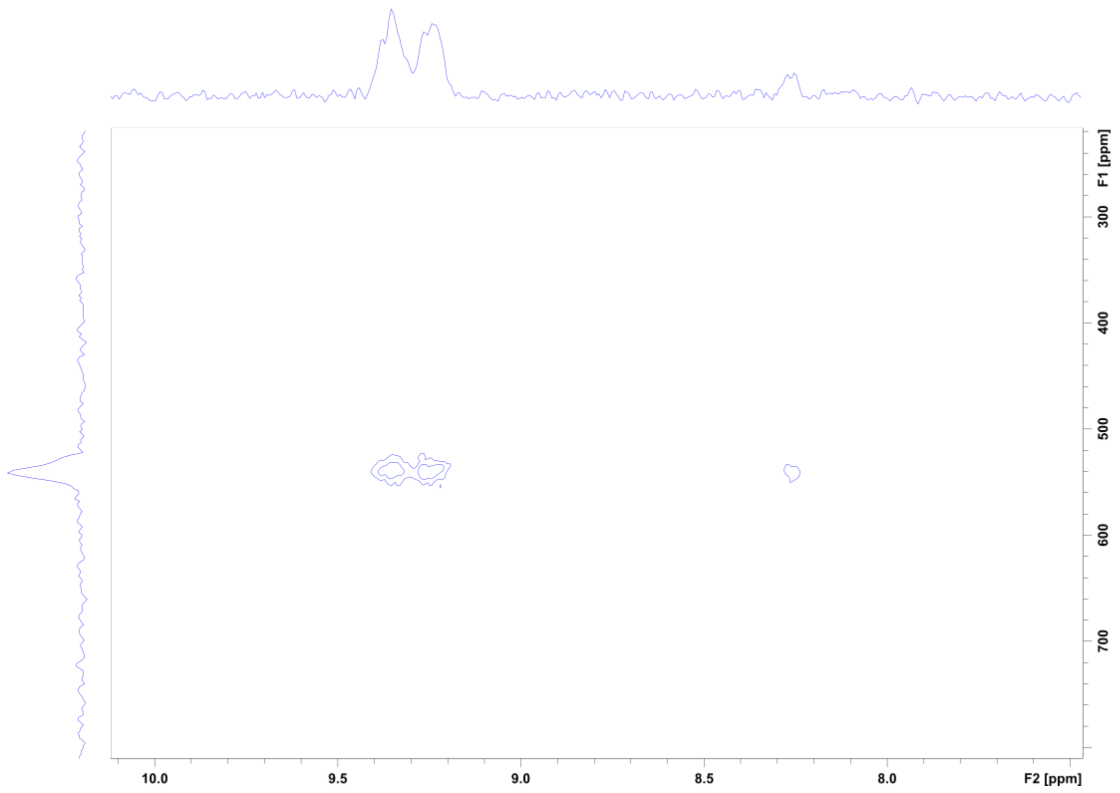

Figure S6. The ^{1}H-^{195}Pt HMQC NMR spectrum of $[Pt(Phen)(SSDACH)(Biotin)(OH)](NO_3)_2$ (**1**) in D_2O at 298 K.

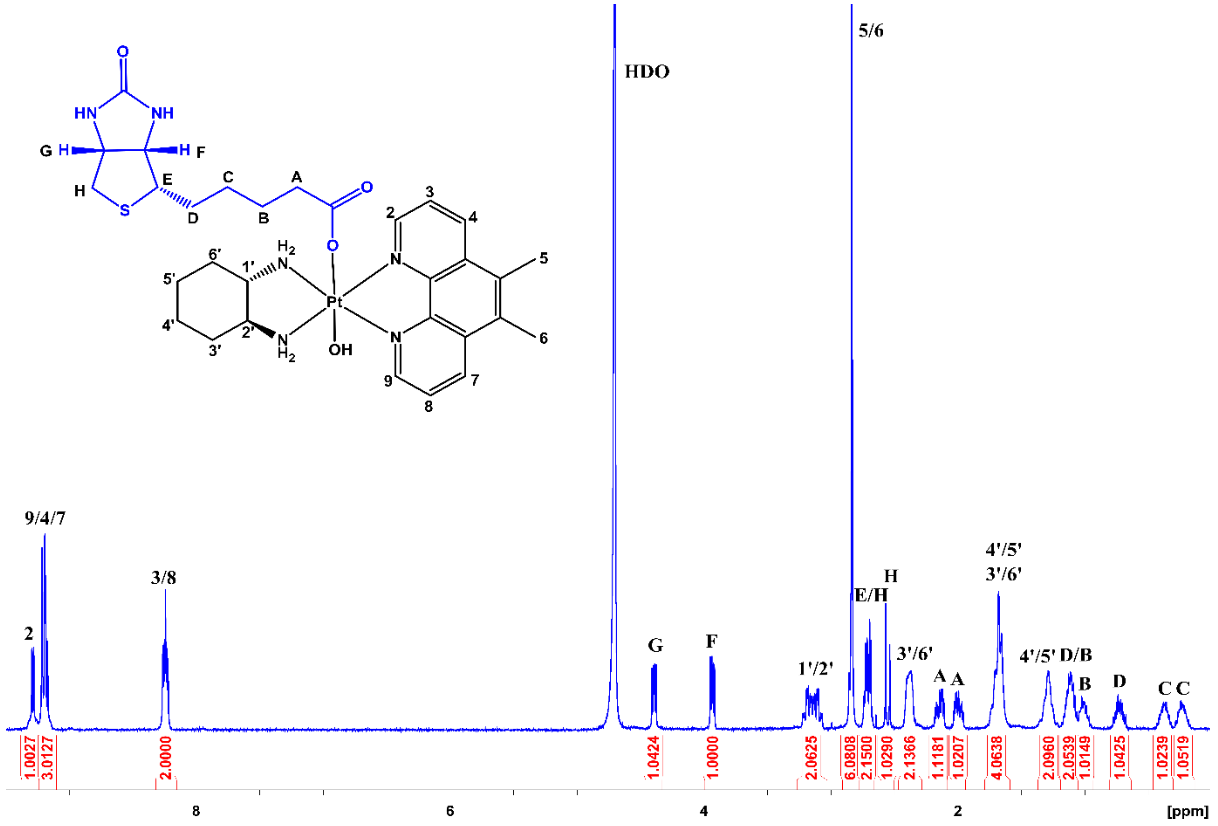

Figure S7. The ^{1}H NMR spectrum of $[Pt(56Me_2Phen)(SSDACH)(Biotin)(OH)](NO_3)_2$ (**2**) in D_2O at 298 K. Insert: The chemical structure of **2** with assigned numbering.

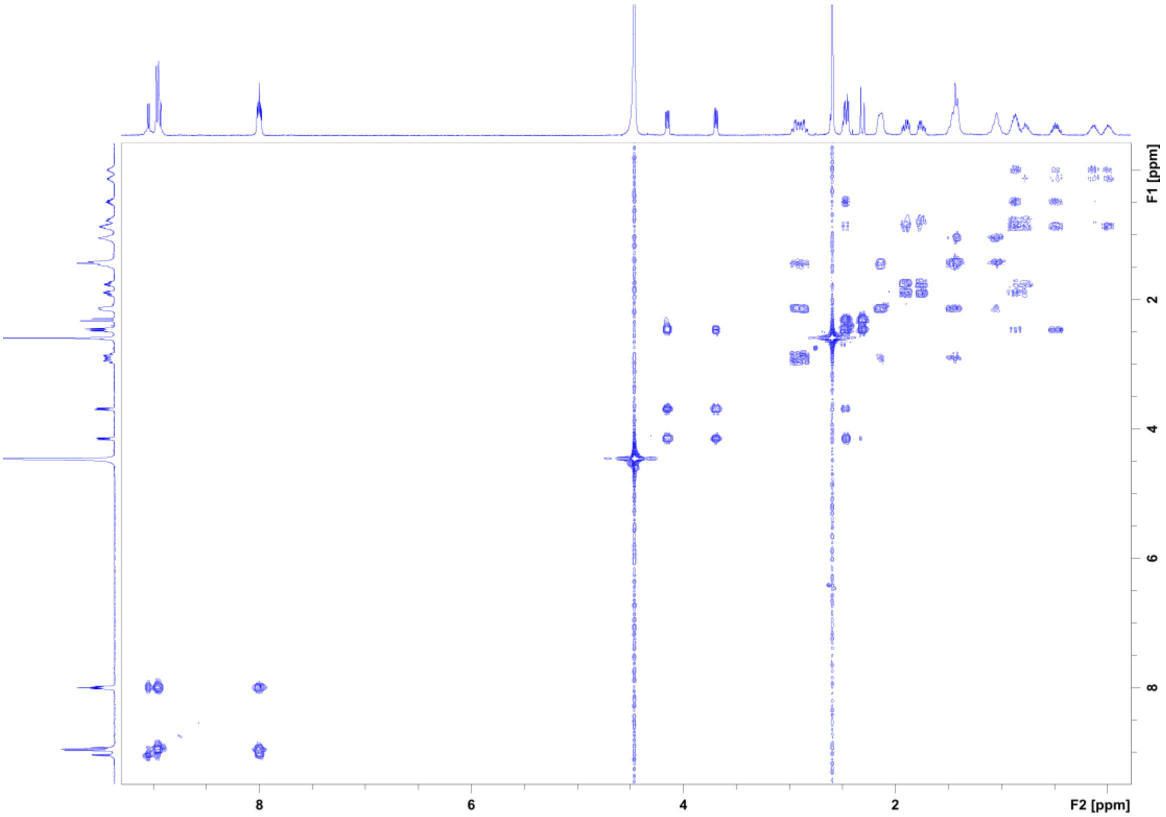

Figure S8. The COSY NMR spectrum of [Pt(56Me_2Phen)(SSDACH)(Biotin)(OH)](NO_3)$_2$ (**2**) in D_2O at 298 K.

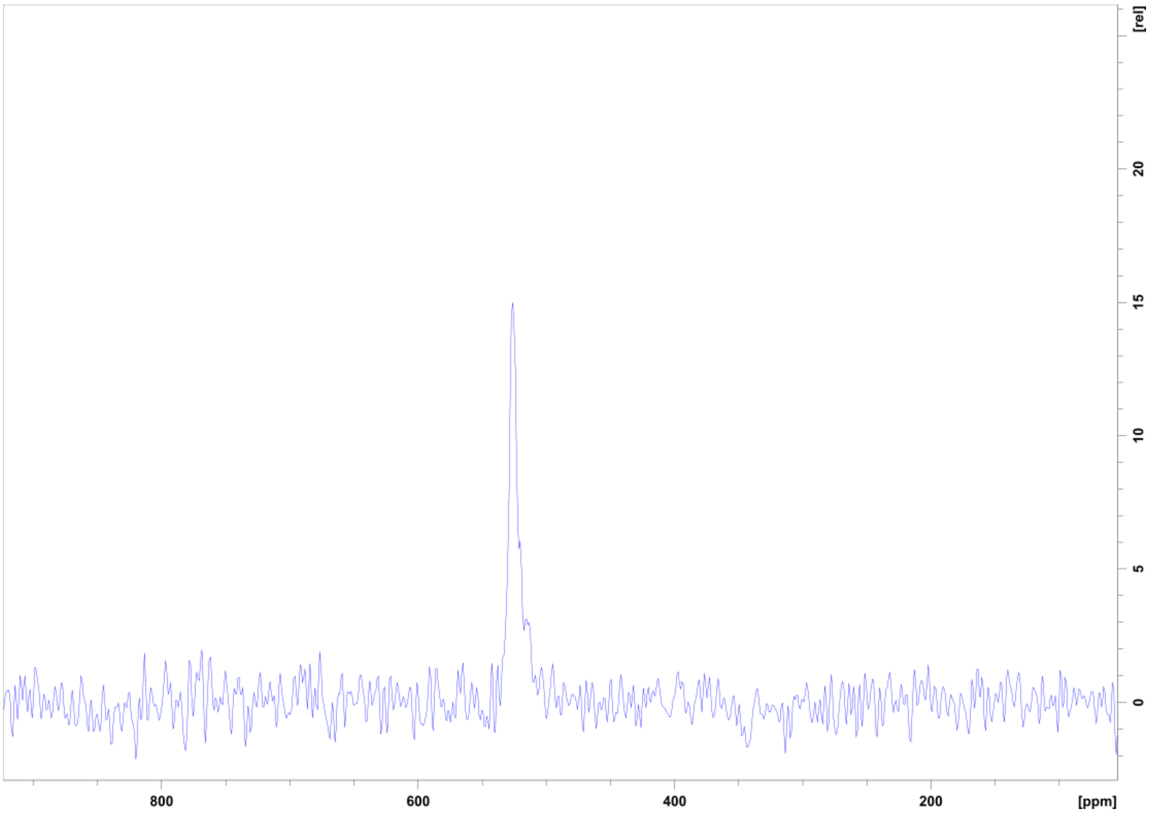

Figure S9. The ^{195}Pt NMR spectrum of [Pt(56Me_2Phen)(SSDACH)(Biotin)(OH)](NO_3)$_2$ (**2**) in D_2O at 298 K, showing a peak at 526 ppm.

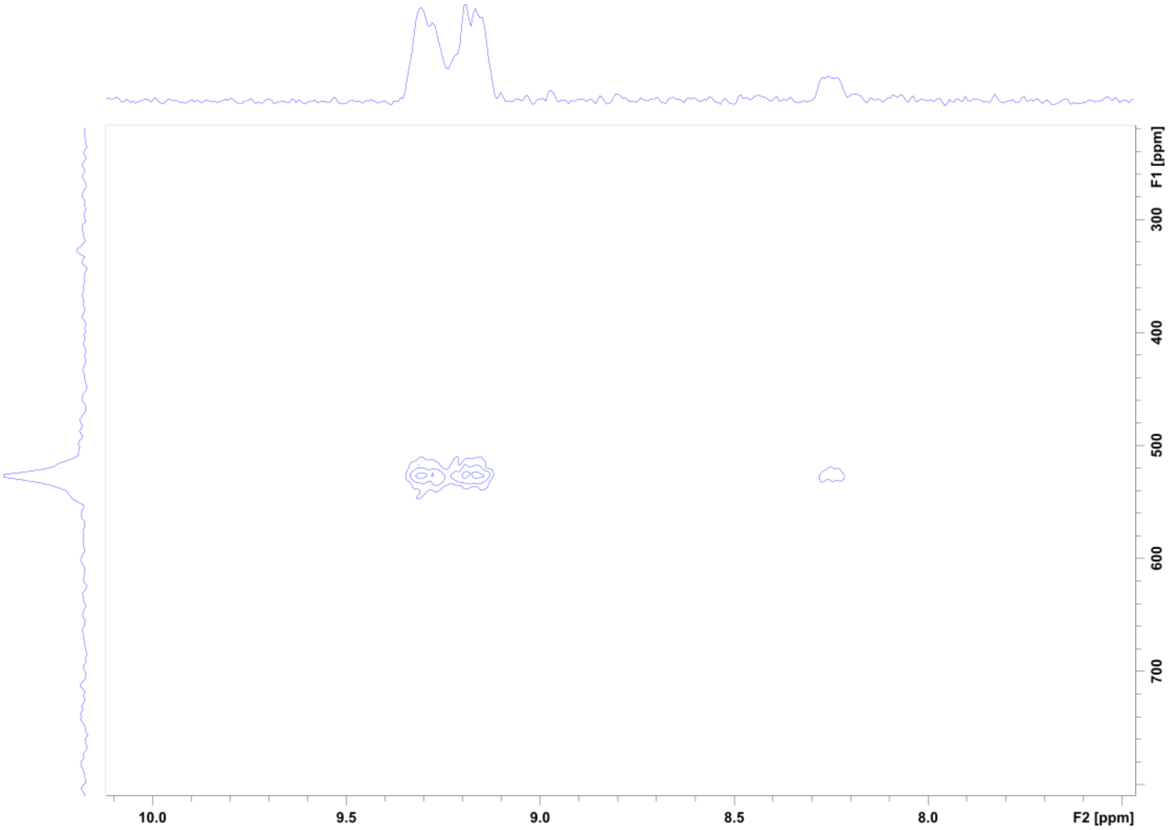

Figure S10. The ^{1}H-^{195}Pt HMQC NMR spectrum of $[Pt(56Me_2Phen)(SSDACH)(Biotin)(OH)](NO_3)_2$ (**2**) in D_2O at 298 K.

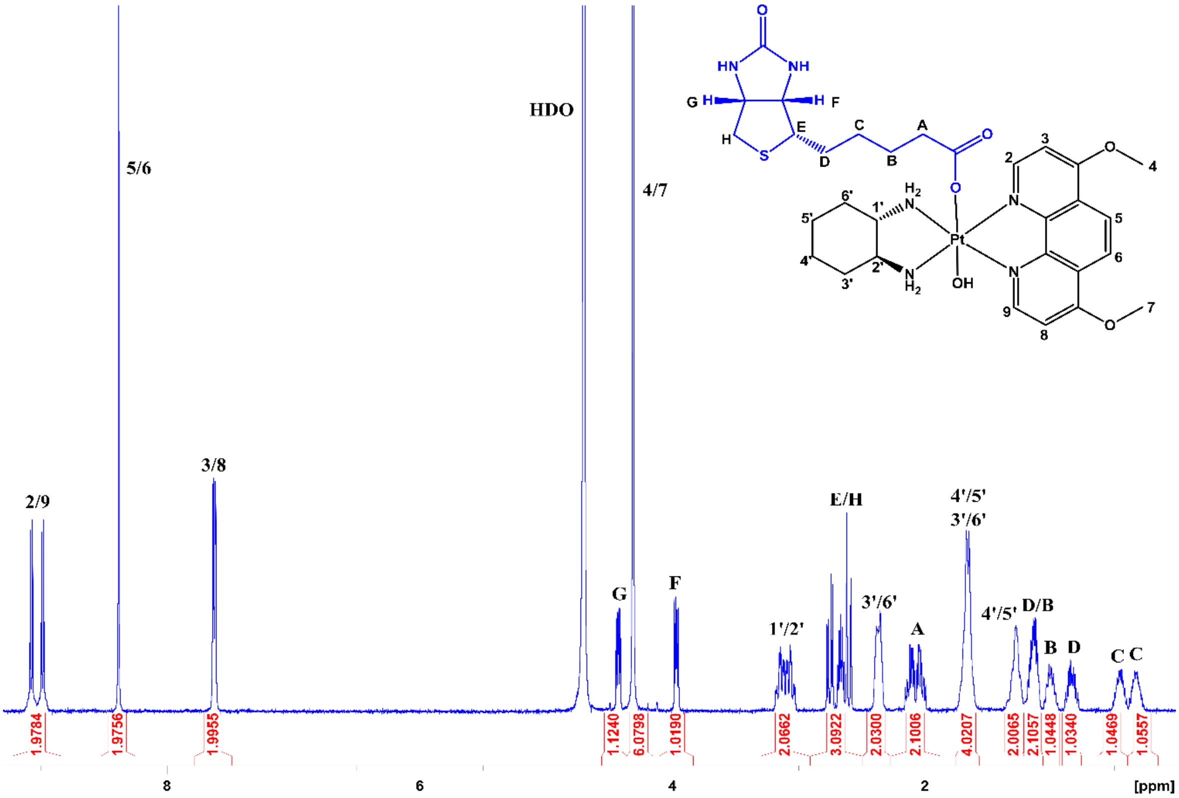

Figure S11. The ^{1}H NMR spectrum of $[Pt(47O_2Me_2Phen)(SSDACH)(Biotin)(OH)](NO_3)_2$ (**3**) in D_2O at 298 K. Insert: The chemical structure of **3** with assigned numbering.

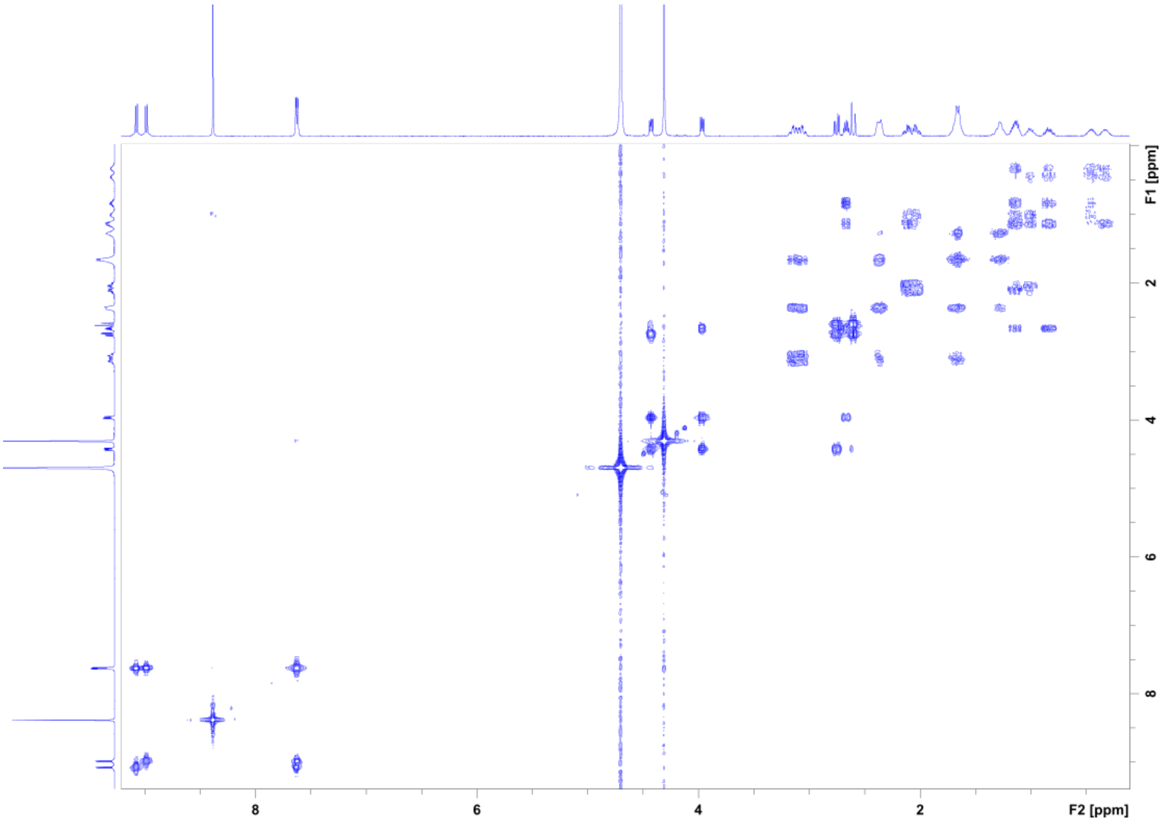

Figure S12. The COSY NMR spectrum of [Pt(47O$_2$Me$_2$Phen)(SSDACH)(Biotin)(OH)](NO$_3$)$_2$ (**3**) in D$_2$O at 298 K.

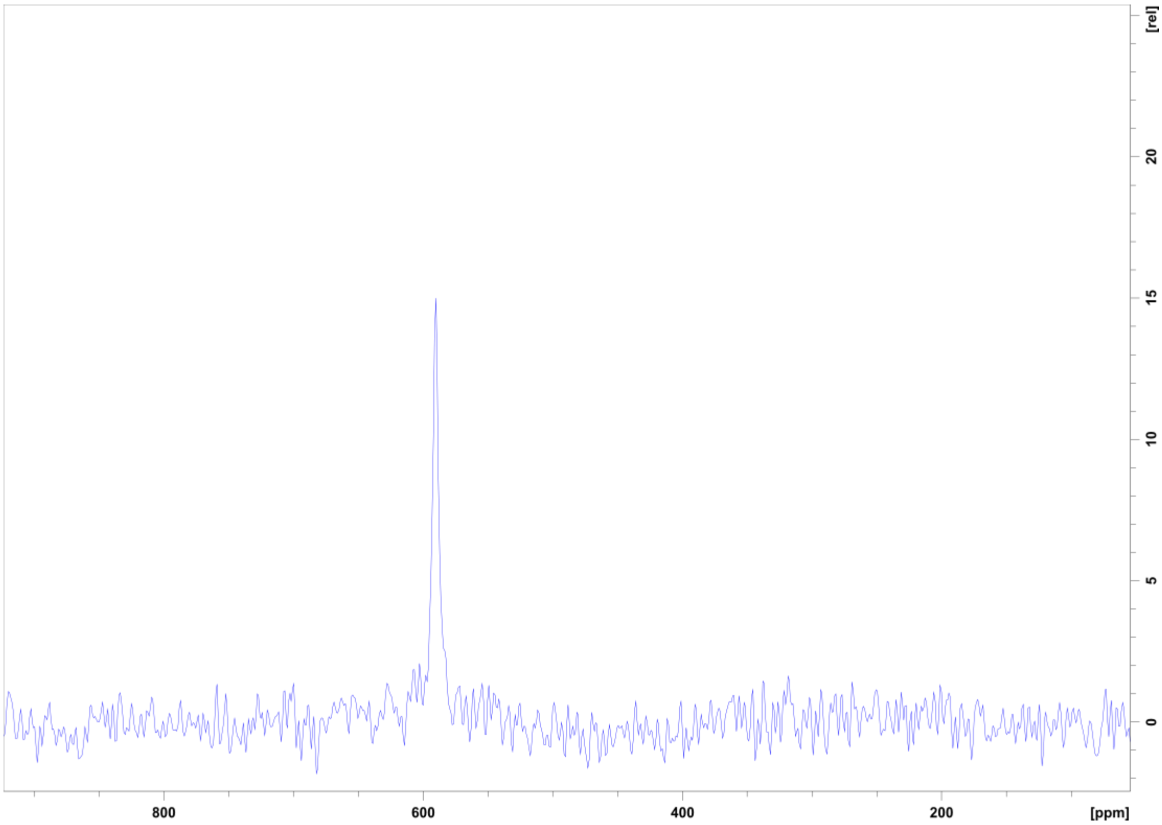

Figure S13. The ^{195}Pt NMR spectrum of [Pt(47O$_2$Me$_2$Phen)(SSDACH)(Biotin)(OH)](NO$_3$)$_2$ (**3**) in D$_2$O at 298 K, showing a peak at 590 ppm.

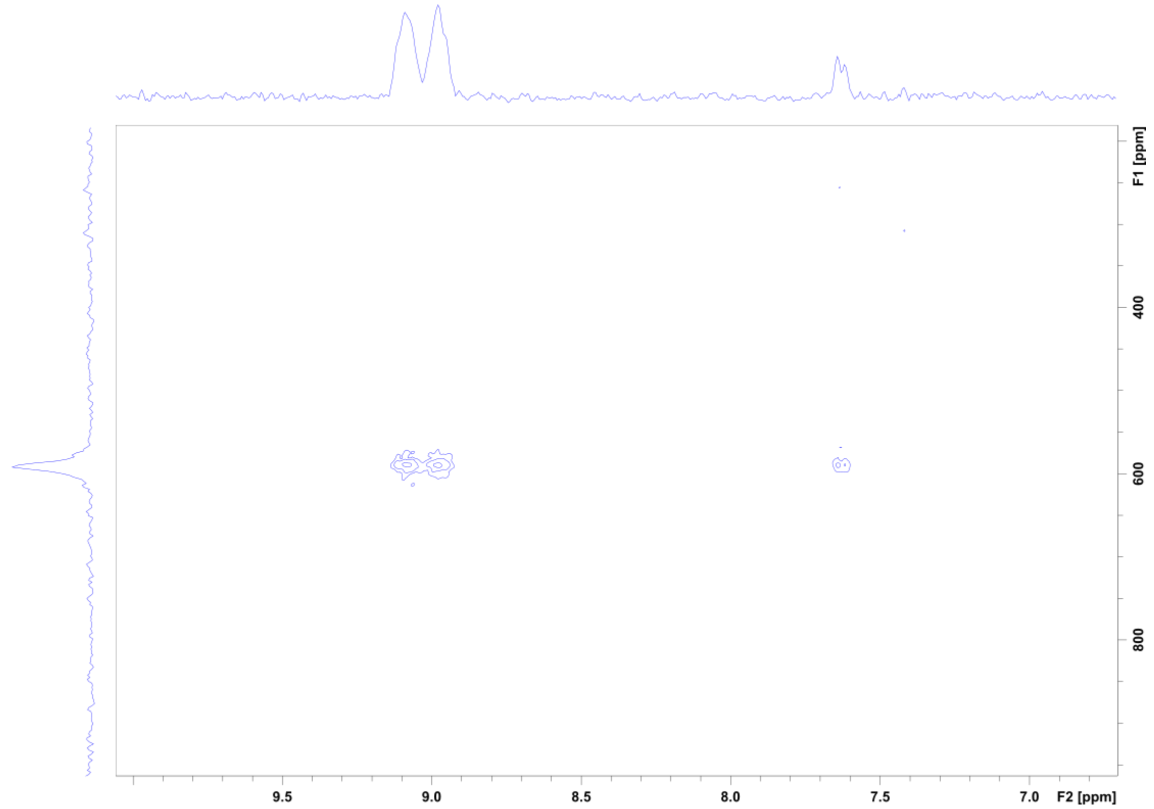

Figure S14. The ^{1}H-^{195}Pt HMQC NMR spectrum of [Pt(47O2Me2Phen)(SSDACH)(Biotin)(OH)]$(NO_3)_2$ (**3**) in D_2O at 298 K.

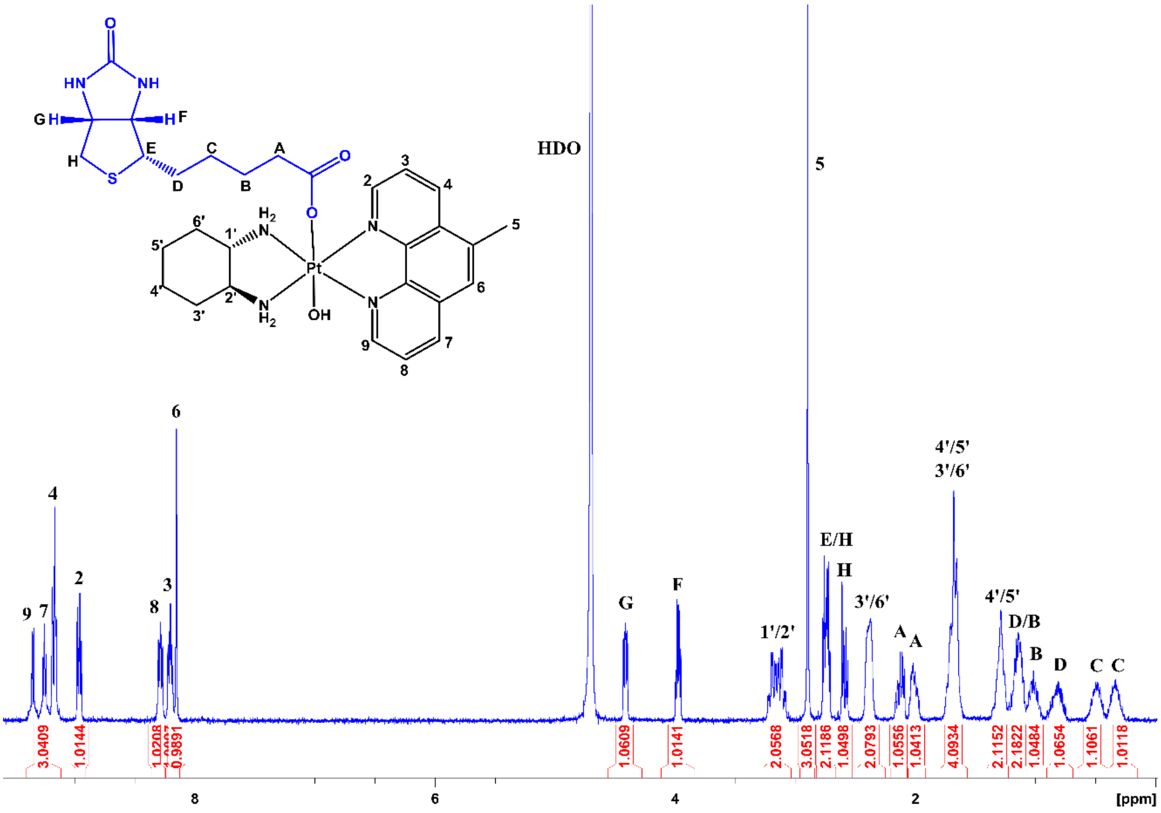

Figure S15. The ^{1}H NMR spectrum of [Pt(5MePhen)(SSDACH)(Biotin)(OH)]$(NO_3)_2$ (**4**) in D_2O at 298 K. Insert: The chemical structure of **4** with assigned numbering.

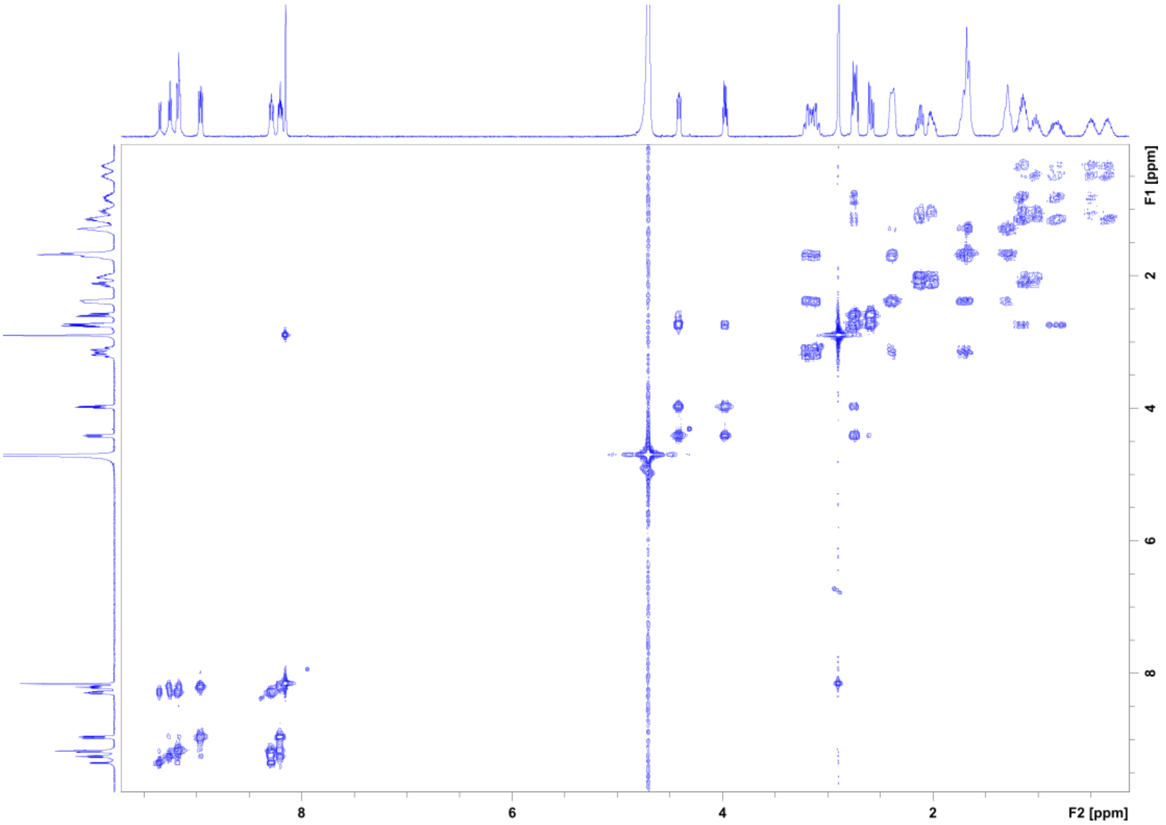

Figure S16. The COSY NMR spectrum of [Pt(5MePhen)(SSDACH)(Biotin)(OH)]$(NO_3)_2$ (**4**) in D_2O at 298 K.

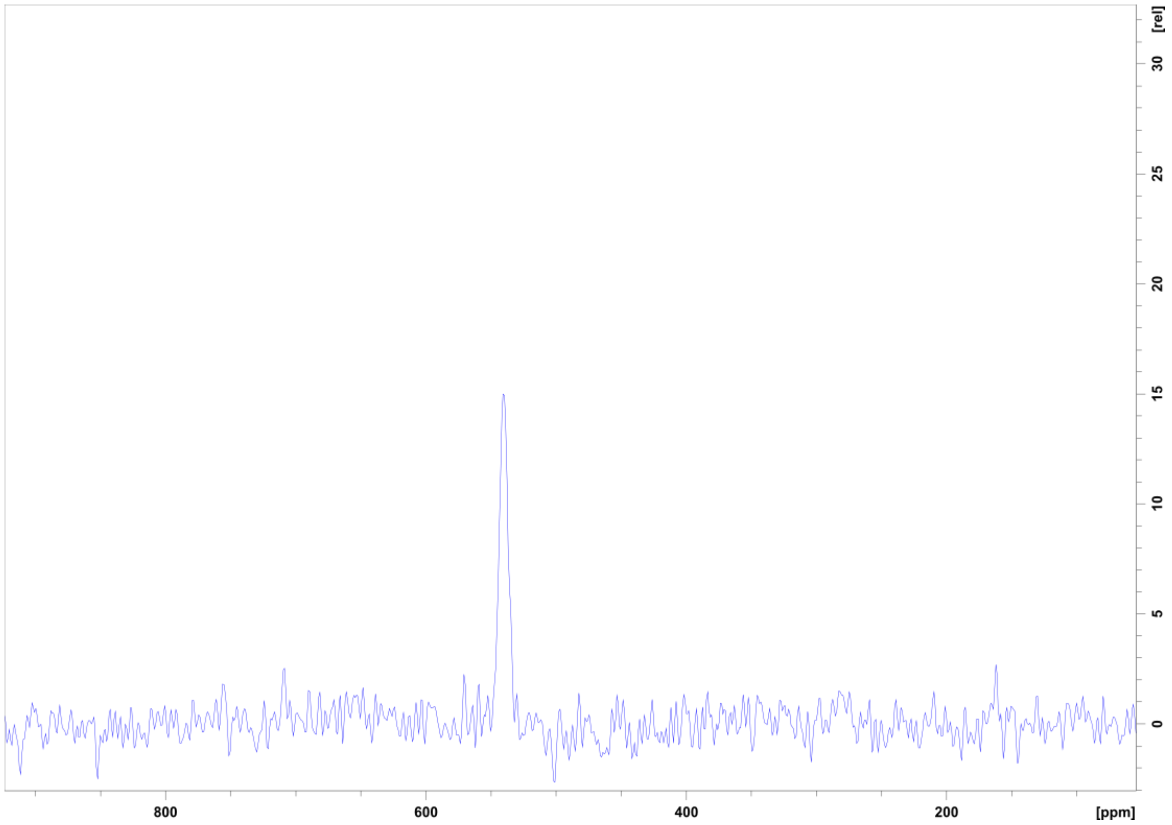

Figure S17. The ^{195}Pt NMR spectrum of [Pt(5MePhen)(SSDACH)(Biotin)(OH)]$(NO_3)_2$ (**4**) in D_2O at 298 K, showing a peak at 540 ppm.

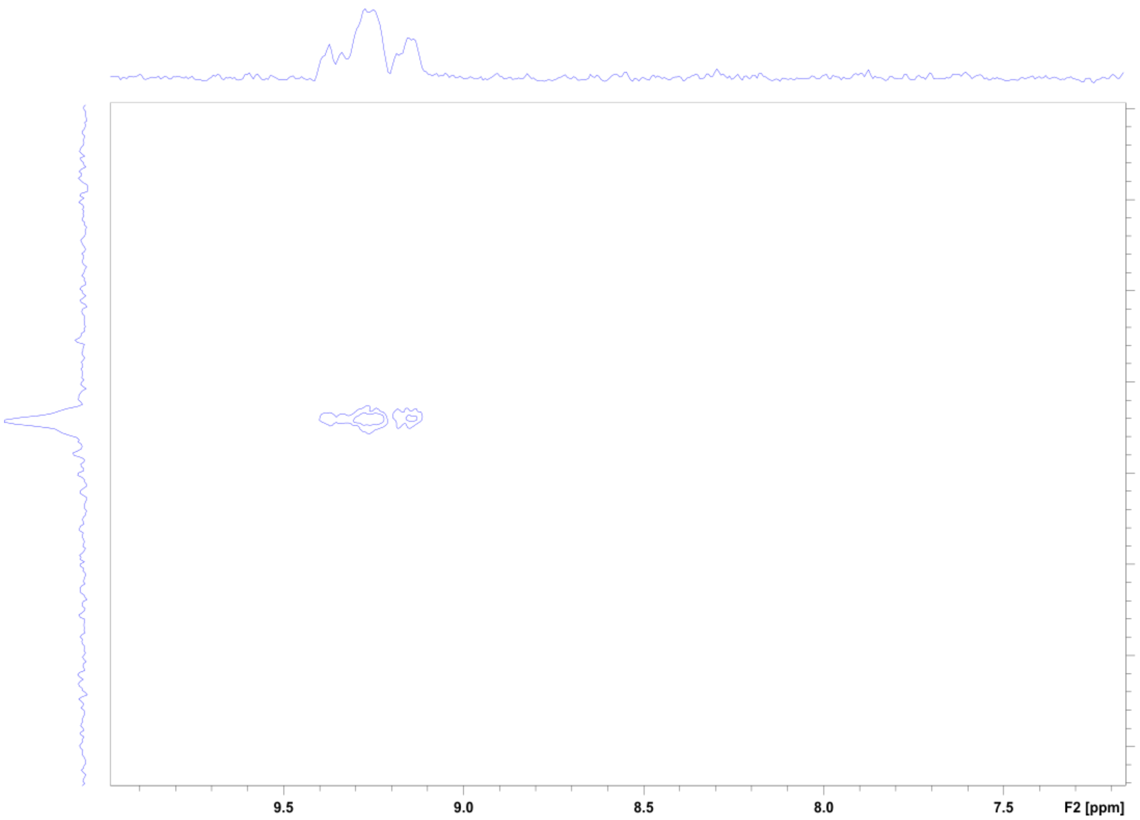

Figure S18. The ^{1}H-^{195}Pt HMQC NMR spectrum of [Pt(5MePhen)(SSDACH)(Biotin)(OH)]$(NO_3)_2$ (**4**) in D_2O at 298 K.

4. HPLC

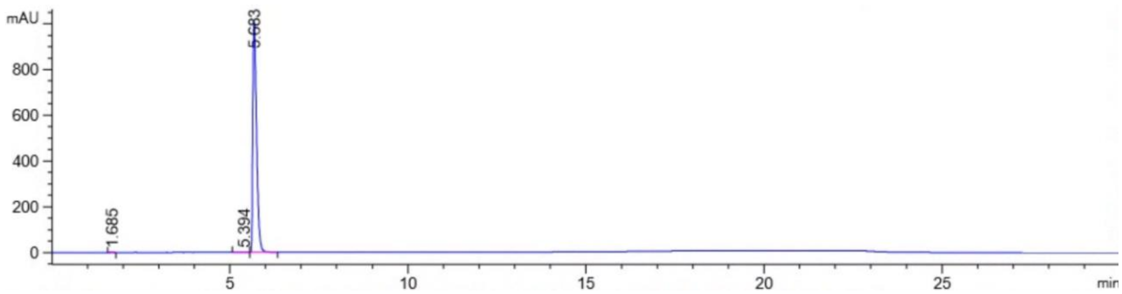

Figure S19. The HPLC chromatogram of [Pt(Phen)(SSDACH)(Biotin)(OH)]$(NO_3)_2$ (**1**), at 254 nm, at a gradient of 0–100% (ACN:H_2O, 9:1) over 15 min.

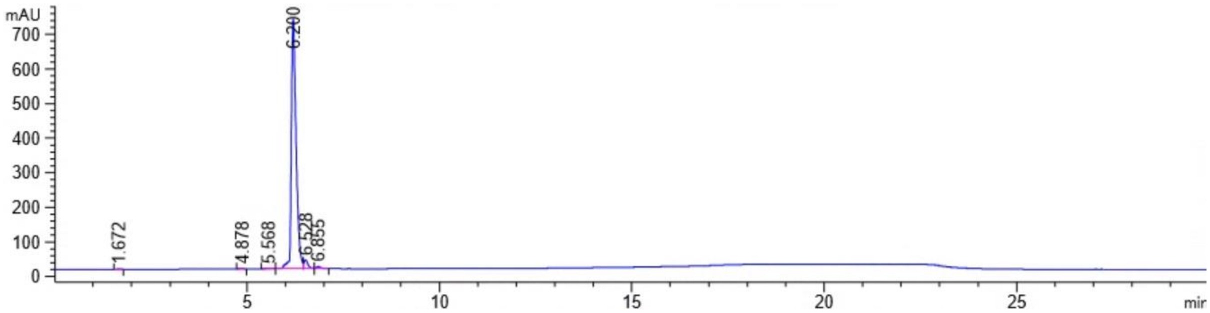

Figure S20. The HPLC chromatogram of [Pt(56Me_2Phen)(SSDACH)(Biotin)(OH)]$(NO_3)_2$ (**2**), at 254 nm, at a gradient of 0–100% (ACN:H_2O, 9:1) over 15 min.

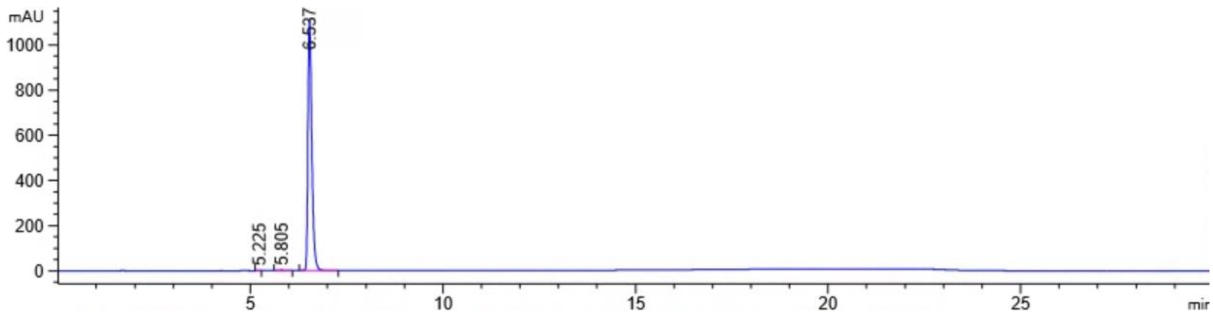

Figure S21. The HPLC chromatogram of [Pt(47O2Me2Phen)(SSDACH)(Biotin)(OH)](NO_3)$_2$ (**3**), at 254 nm, at a gradient of 0–100% (ACN:H_2O, 9:1) over 15 min.

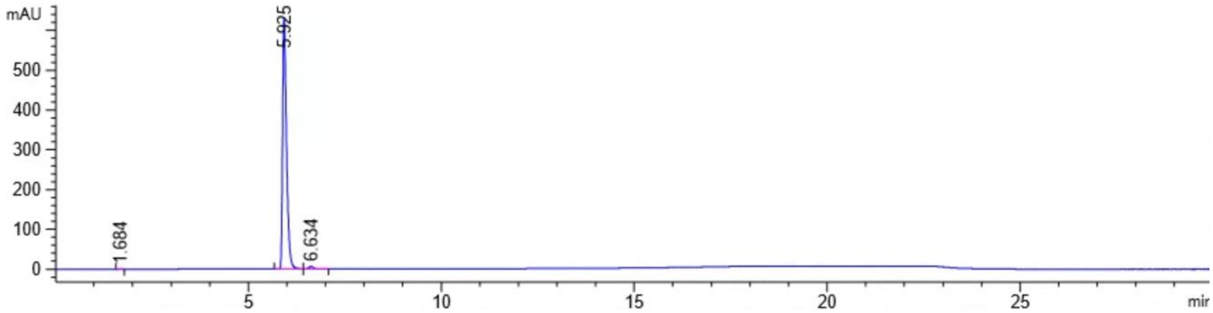

Figure S22. The HPLC chromatogram of [Pt(5MePhen)(SSDACH)(Biotin)(OH)](NO_3)$_2$ (**4**), at 254 nm, at a gradient of 0–100% (ACN:H_2O, 9:1) over 15 min.

5. ESI-MS

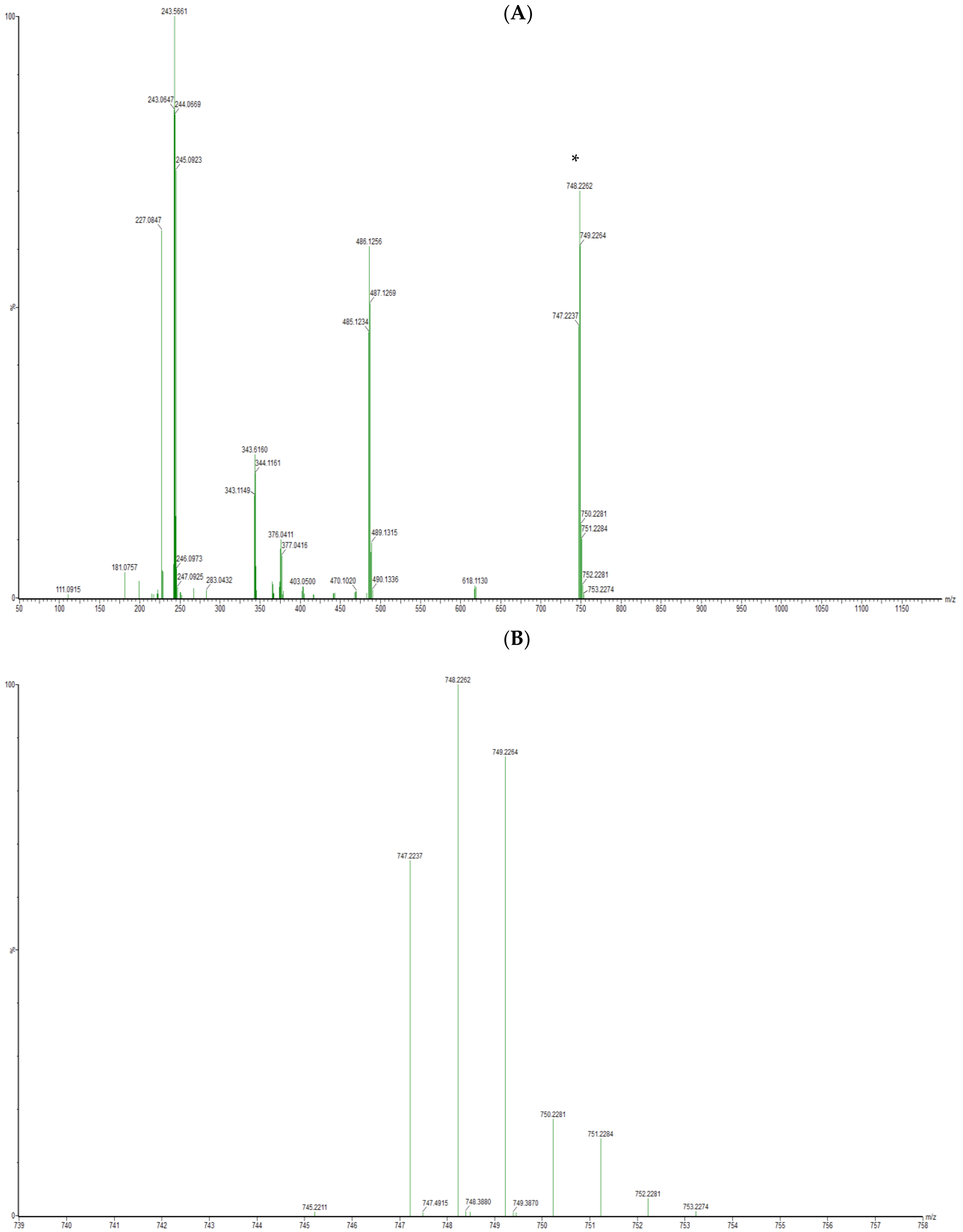

Figure S23. (**A**) Full ESI-MS spectrum of [Pt(Phen)(SSDACH)(Biotin)(OH)]$(NO_3)_2$ (**1**). (**B**) The expanded region of the [M-H]$^+$ peak.

(**A**)

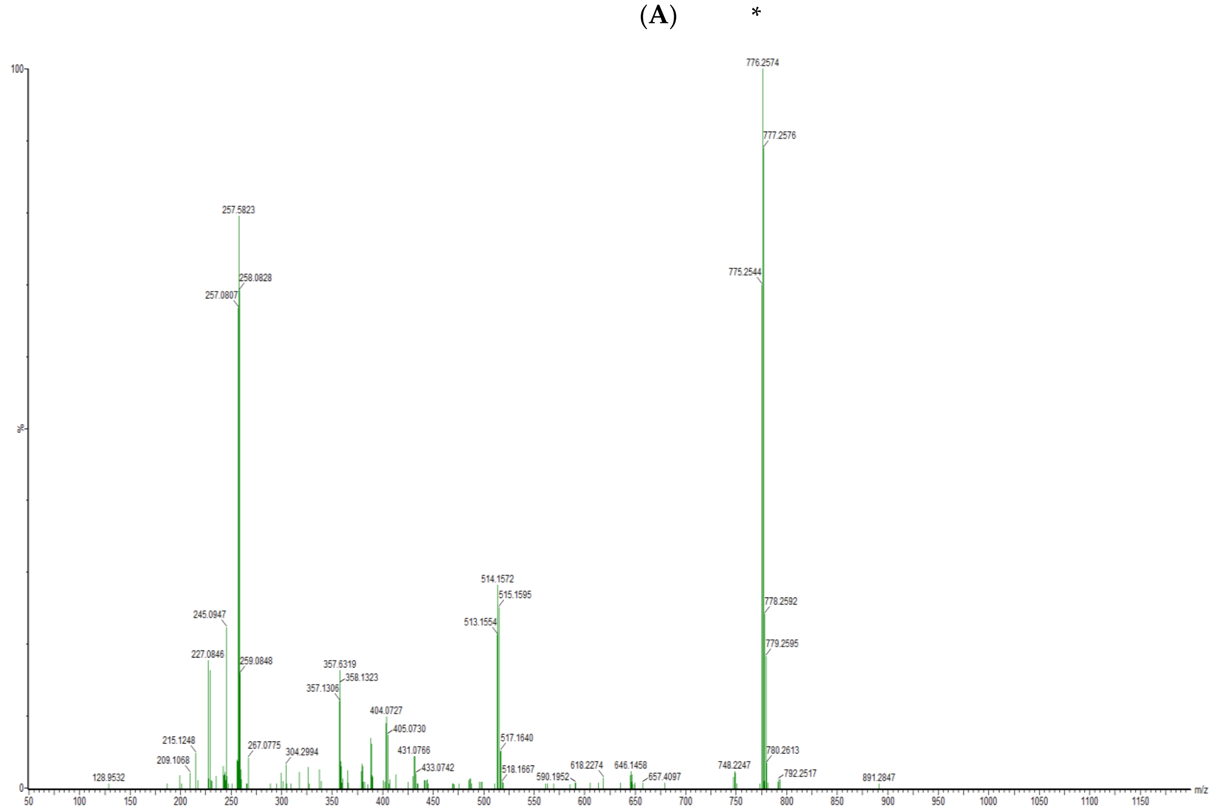

(**B**)

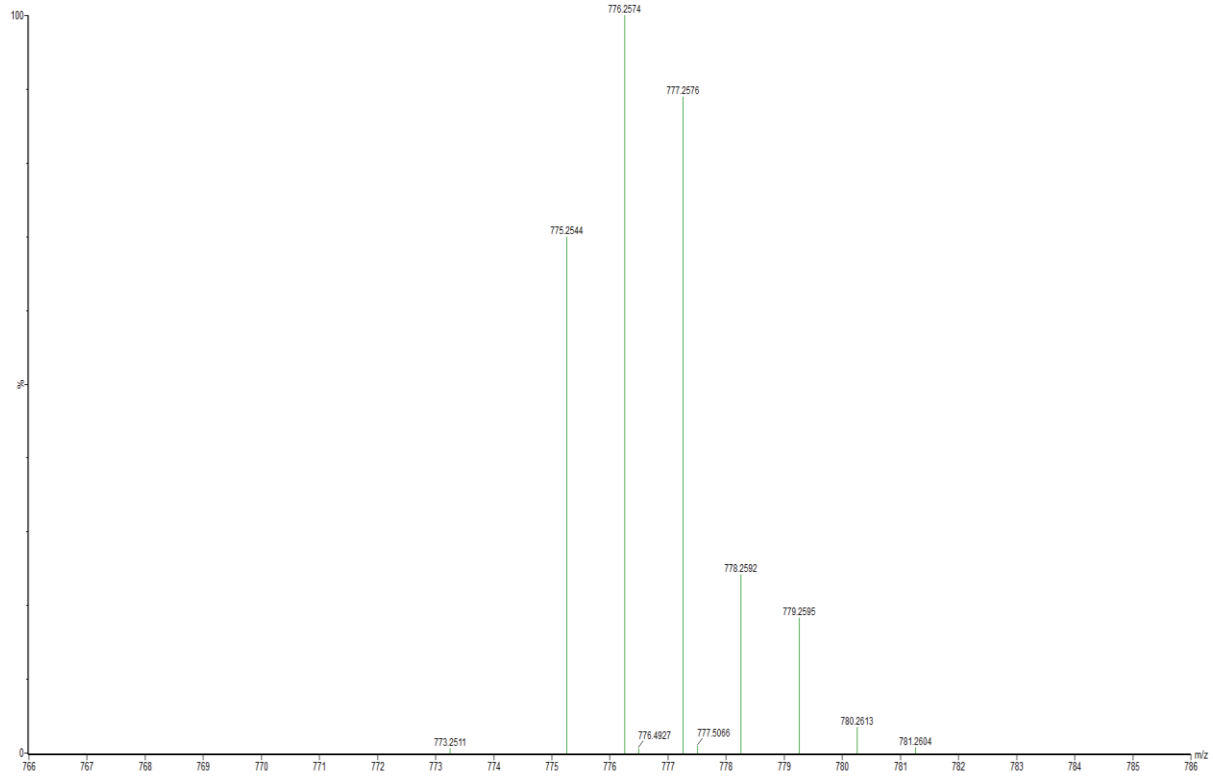

Figure S24. (**A**) Full ESI-MS spectrum of [Pt(56Me_2Phen)(SSDACH)(Biotin)(OH)]$(NO_3)_2$ (**2**). (**B**) The expanded region of the $[M-H]^+$ peak.

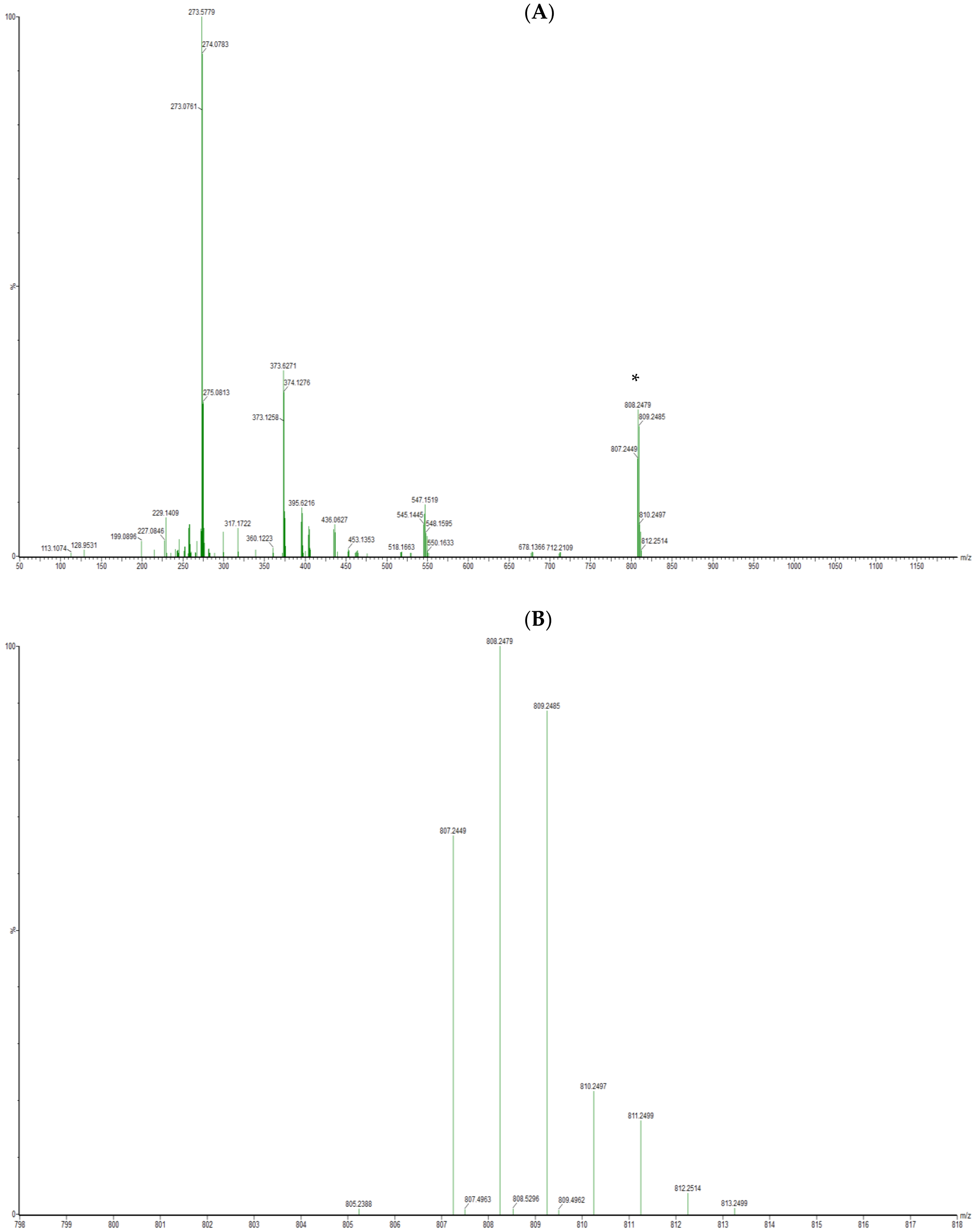

Figure S25. (**A**) Full ESI-MS spectrum of [Pt(47O$_2$Me$_2$Phen)(SSDACH)(Biotin)(OH)](NO$_3$)$_2$ (**3**). (**B**) The expanded region of the [M-H]$^+$ peak.

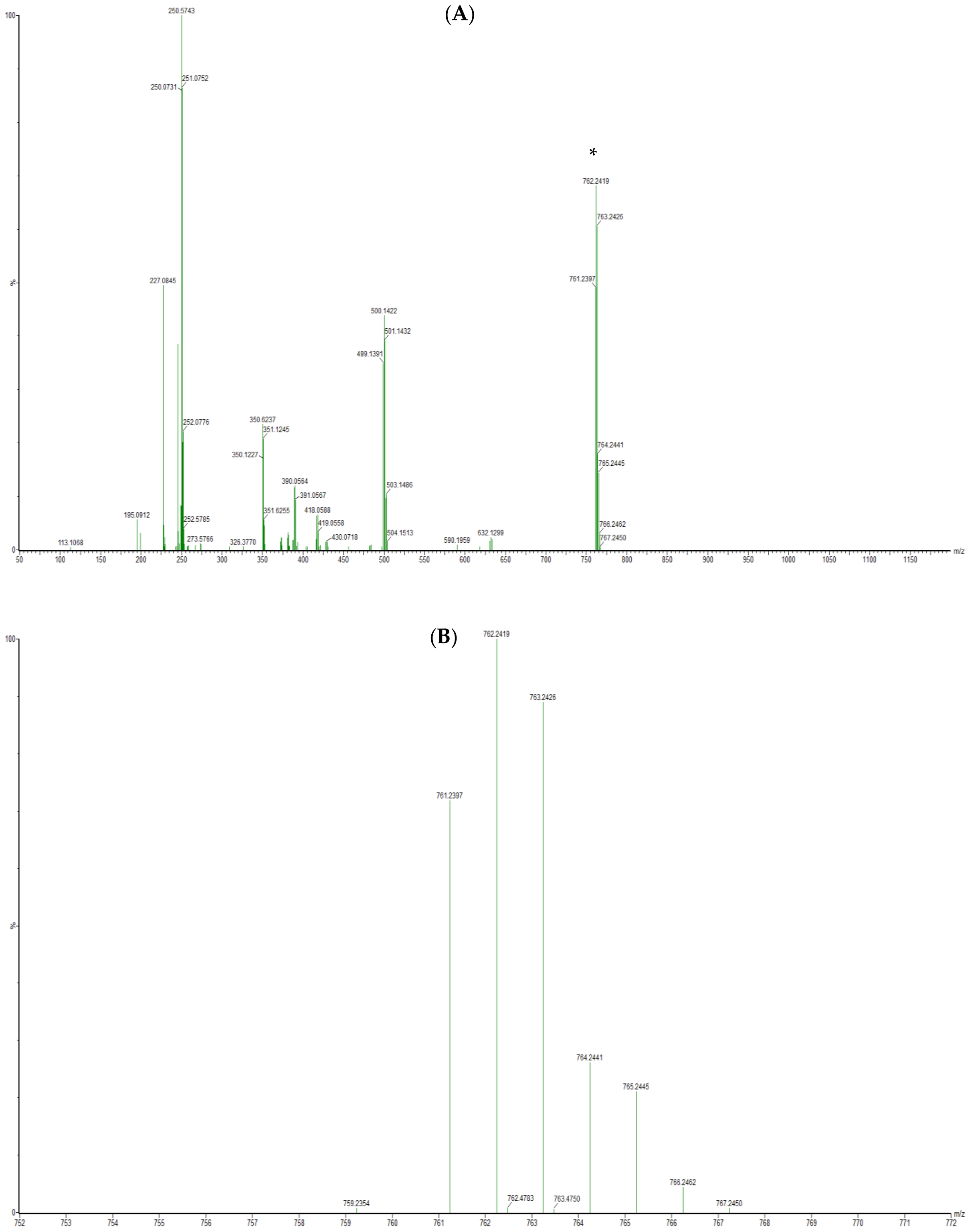

Figure S26. (**A**) Full ESI-MS spectrum of [Pt(5MePhen)(SSDACH)(Biotin)(OH)]$(NO_3)_2$ (**4**). (**B**) The expanded region of the $[M-H]^+$ peak.

7. Summary of Characterisation Data

Table S3. Summary of characterisation data for **1–4**.

Complex	Molecular Formula	Yield (%)	HRMS-ESI: Calc. $[M-N_2O_6-H]^+$ *m/z* (found).	Electronic spectrum λ_{max} nm (ε/M^{-1} cm^{-1})	CD λ_{max} nm (mdeg mol L^{-1})	HPLC purity (%)
**1**	$C_{28}H_{38}N_8O_{10}PtS$	66	748.2245 (748.2262)	204 (70,400 ± 180), 279 (25,300 ± 70), 306 (6200 ± 85)	212 (-2.8), 276 (-0.4)	99.7
**2**	$C_{30}H_{42}N_8O_{10}PtS$	64	776.2558 (776.2574)	206 (75,900 ± 360), 290 (26,800 ± 170), 318 (6700 ± 30)	212 (-2.6), 243 (-0.6), 256 (0.1), 286 (-0.6)	95.7
**3**	$C_{30}H_{42}N_8O_{12}PtS$	62	808.2456 (808.2479)	194 (110,400 ± 190), 212 (103,200 ± 130), 267 (60,100 ± 130), 280 (38,100 ± 320), 347 (8200 ± 15)	214 (-2.7), 247 (0.1), 268 (-0.5), 305 (0.5)	99.6
**4**	$C_{29}H_{40}N_8O_{10}PtS$	65	762.2401 (762.2419)	206 (72,400 ± 310), 284 (25,800 ± 160), 312 (6500 ± 50)	212 (-1.8), 242 (-0.3), 251 (0.3), 284 (-0.3)	98.8

8. UV Spectra

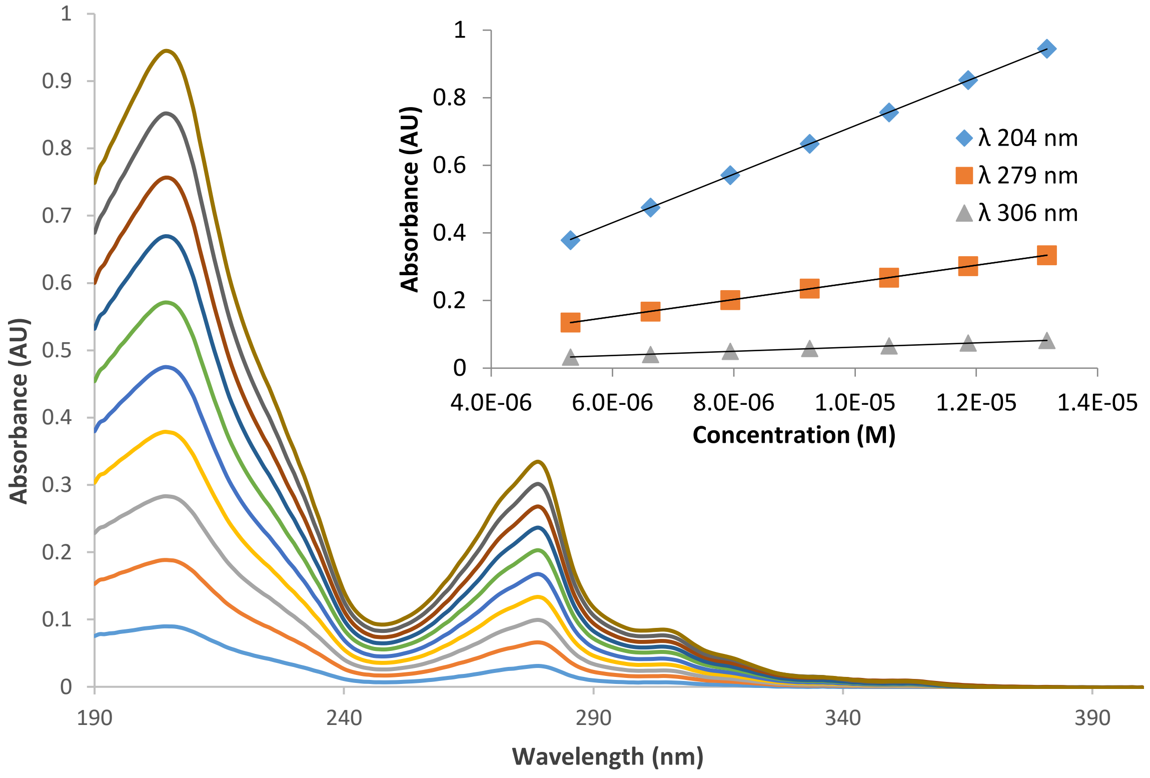

Figure S27. The UV spectrum of a replicate of [Pt(Phen)(SSDACH)(Biotin)(OH)]$(NO_3)_2$ (**1**) in water.

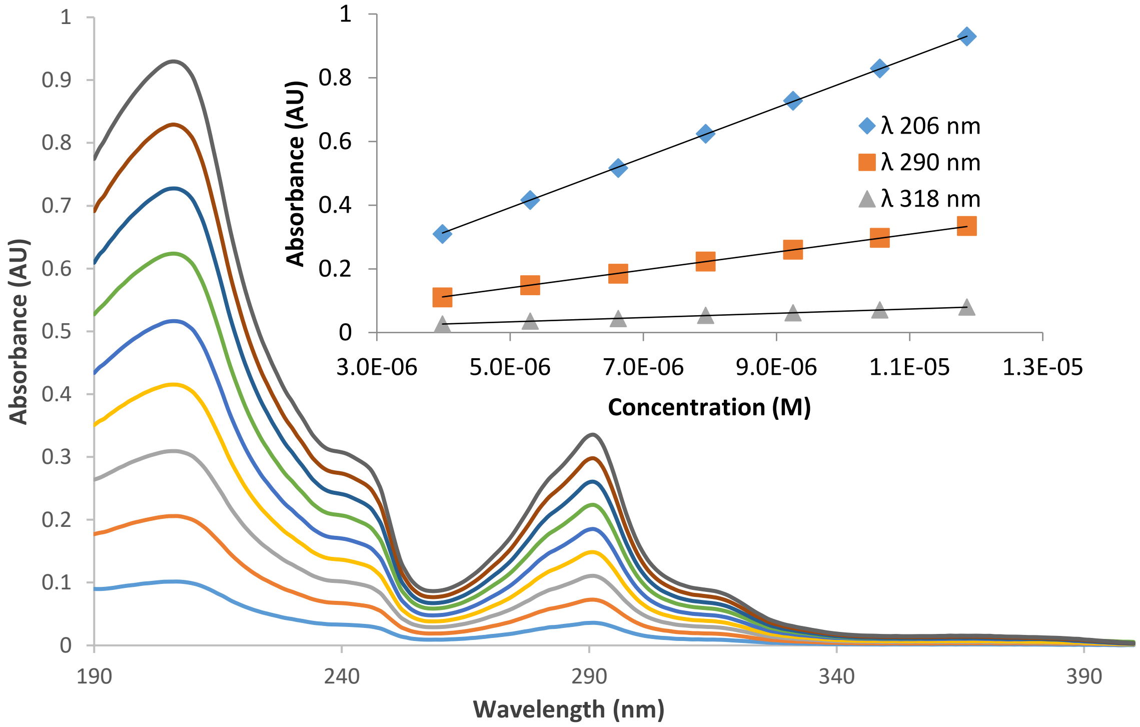

Figure S28. The UV spectrum of a replicate of [Pt(56Me_2Phen)(SSDACH)(Biotin)(OH)]$(NO_3)_2$ (**2**) in water.

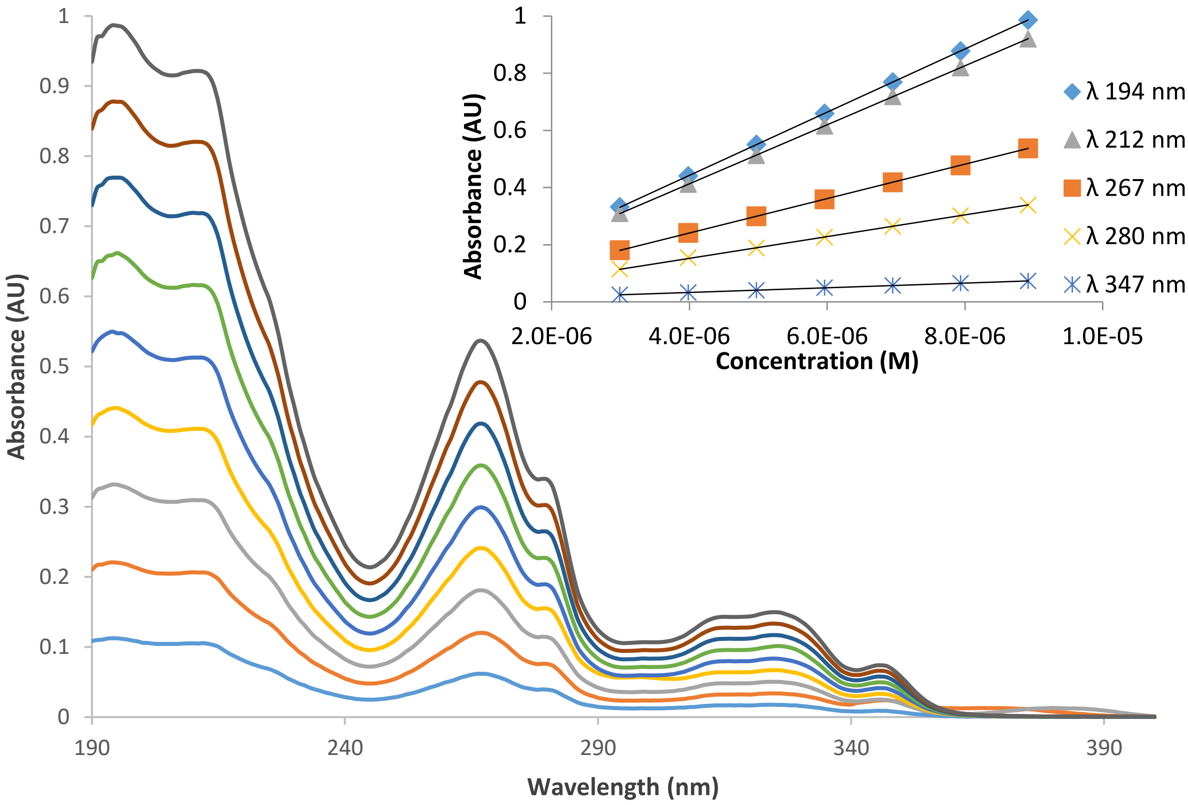

Figure S29. The UV spectrum of a replicate of [Pt(47O$_2$Me$_2$Phen)(SSDACH)(Biotin)(OH)](NO$_3$)$_2$ (**3**) in water.

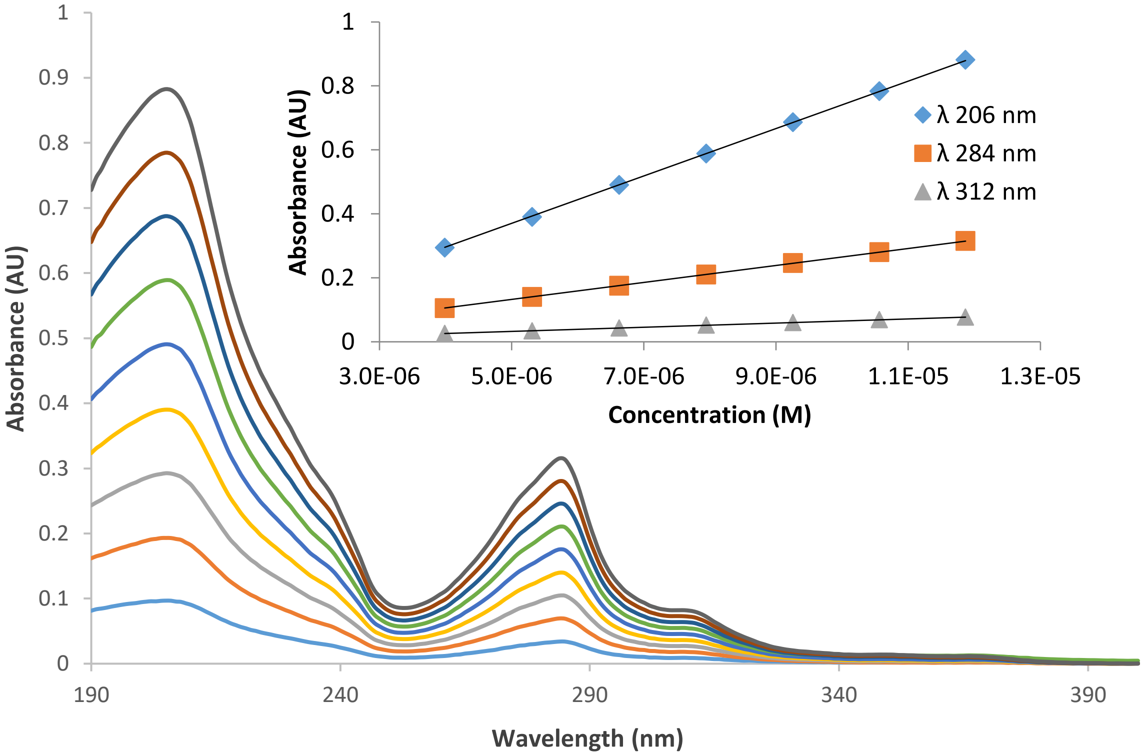

Figure S30. The UV spectrum of a replicate of [Pt(5MePhen)(SSDACH)(Biotin)(OH)](NO$_3$)$_2$ (**4**) in water.

9. CD Spectra

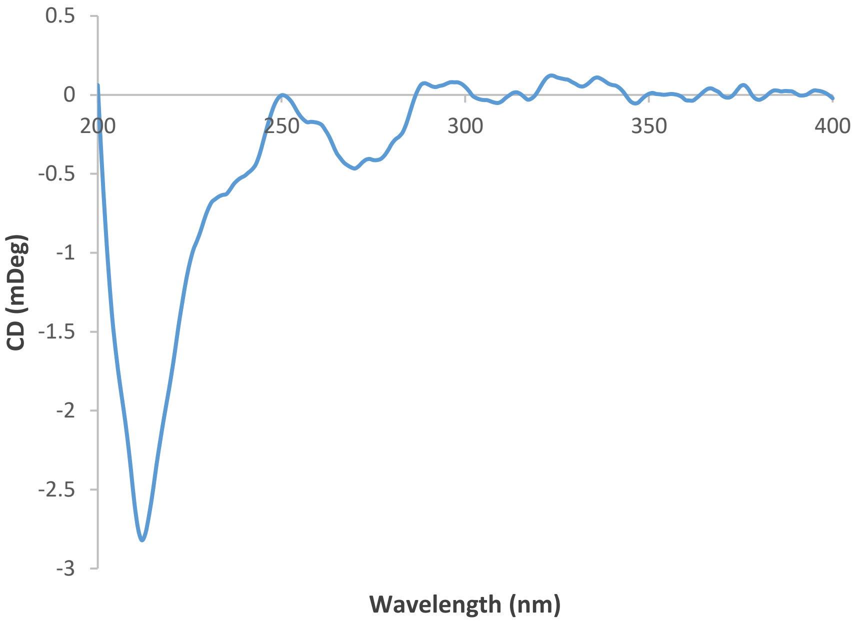

Figure S31. The CD spectrum of [Pt(Phen)(SSDACH)(Biotin)(OH)]$(NO_3)_2$ (**1**) in water. 9pt smoothing applied.

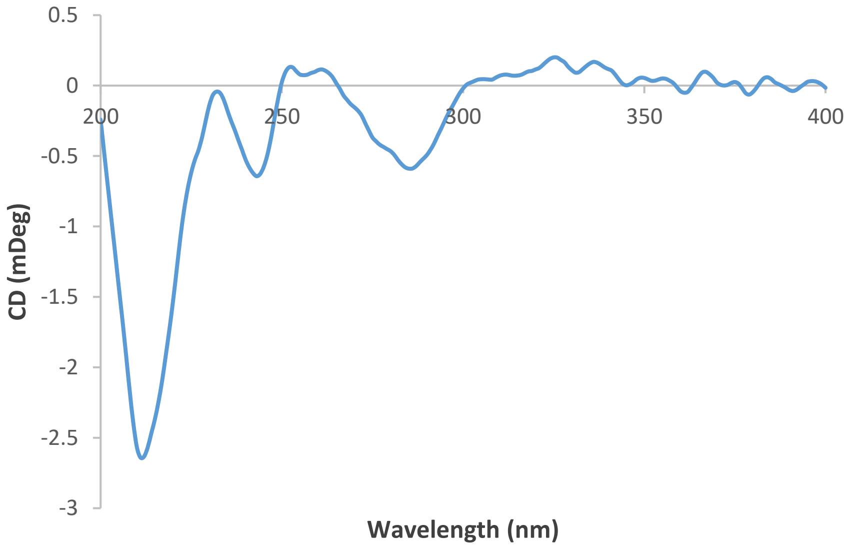

Figure S32. The CD spectrum of [Pt(56Me_2Phen)(SSDACH)(Biotin)(OH)]$(NO_3)_2$ (**2**) in water. 9pt smoothing applied.

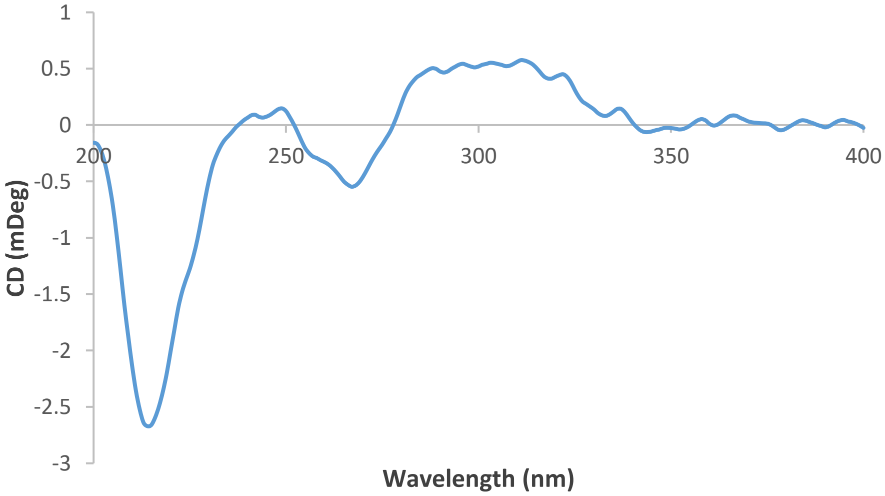

Figure S33. The CD spectrum of [Pt(47O_2Me_2Phen)(SSDACH)(Biotin)(OH)]$(NO_3)_2$ (**3**) in water. 9pt smoothing applied.

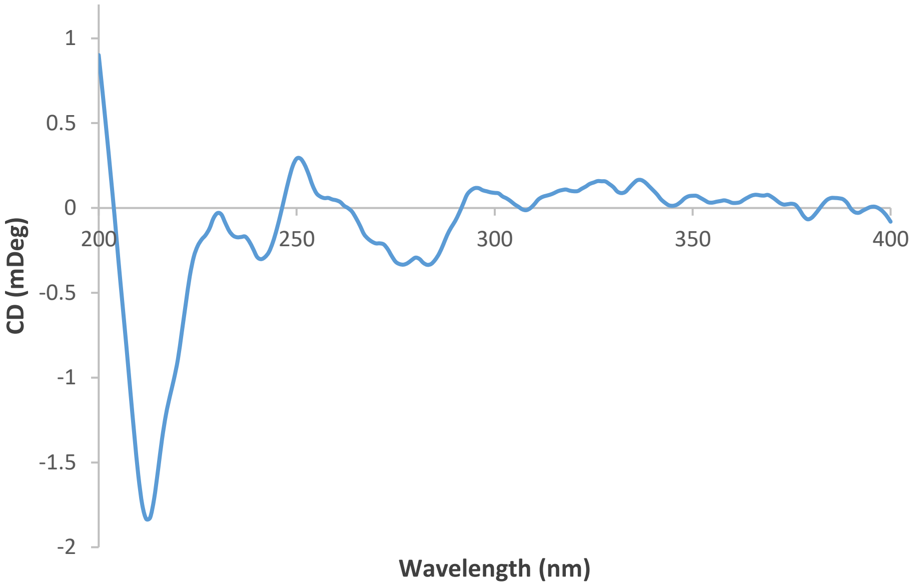

Figure S34. The CD spectrum of [Pt(5MePhen)(SSDACH)(Biotin)(OH)]$(NO_3)_2$ (**4**) in water. 9pt smoothing applied.

11. Reduction Studies

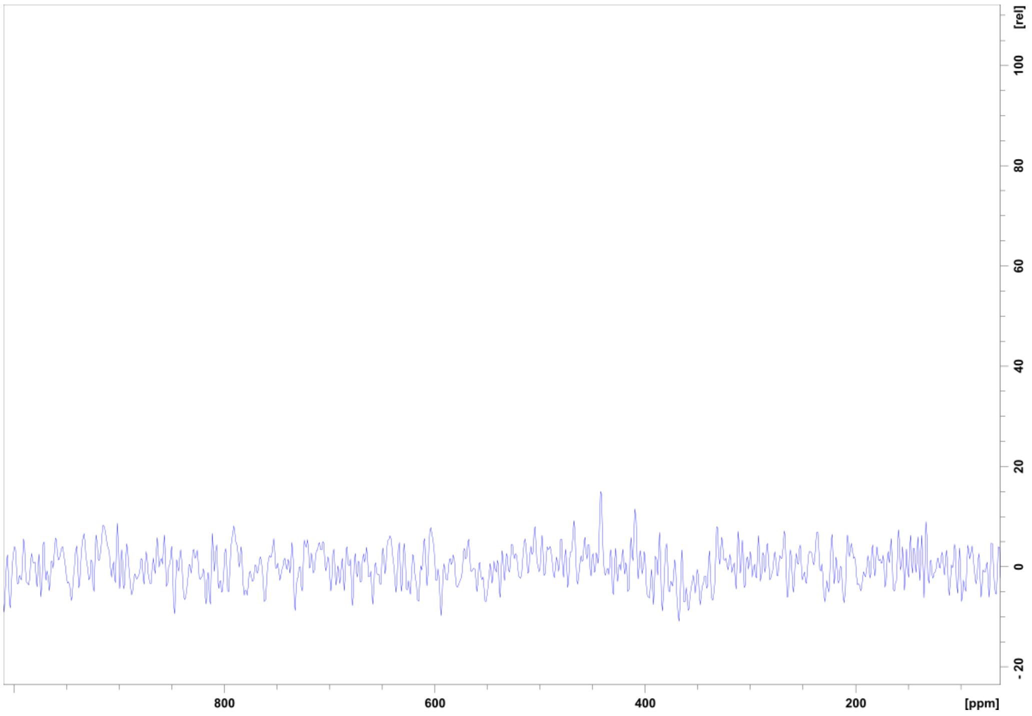

Figure S35. The Pt(IV) region in the ^{195}Pt NMR spectrum of **3** after reduction, showing the disappearance of the peak at 590 ppm.

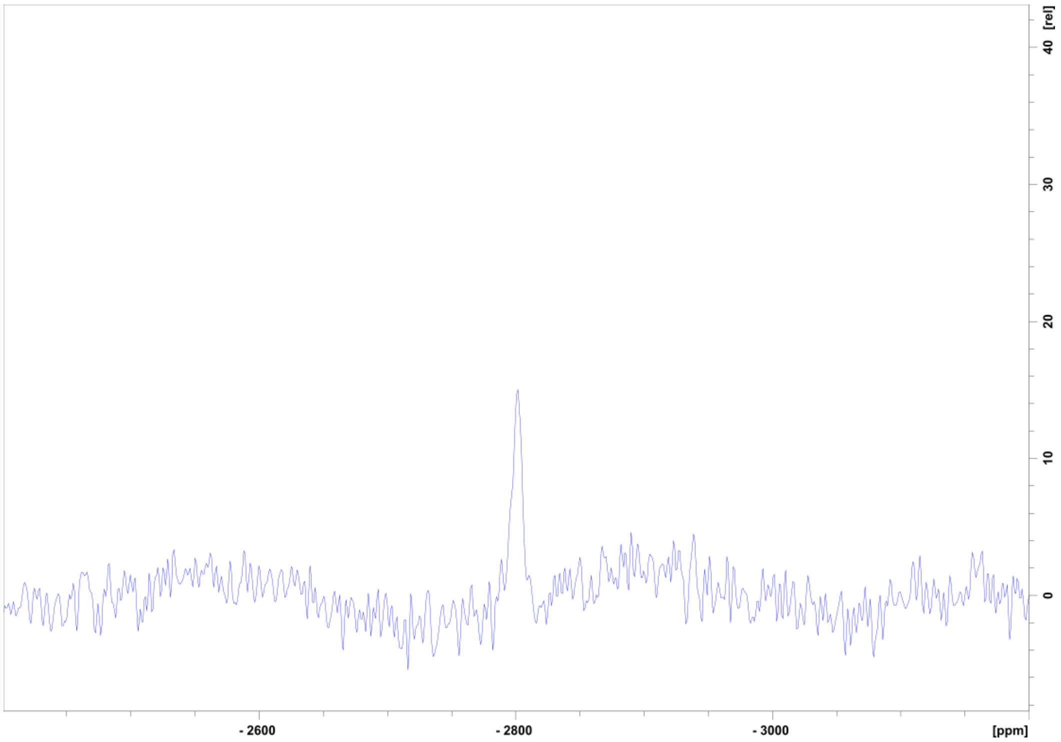

Figure S36. The Pt(II) region in the ^{195}Pt NMR spectrum of **3** after reduction, showing a new peak at -2801 ppm.

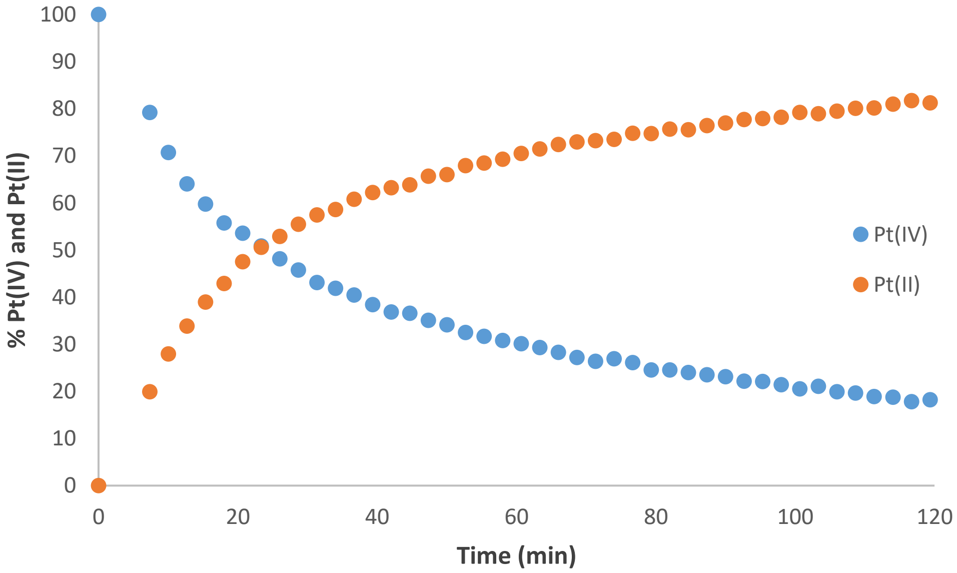

Figure S37. A replicate of the time course reduction study of **3** *via* ^{1}H NMR. The Pt(II) and Pt(IV) resonances are plotted as a percentage.

12. Lipophilicity Studies

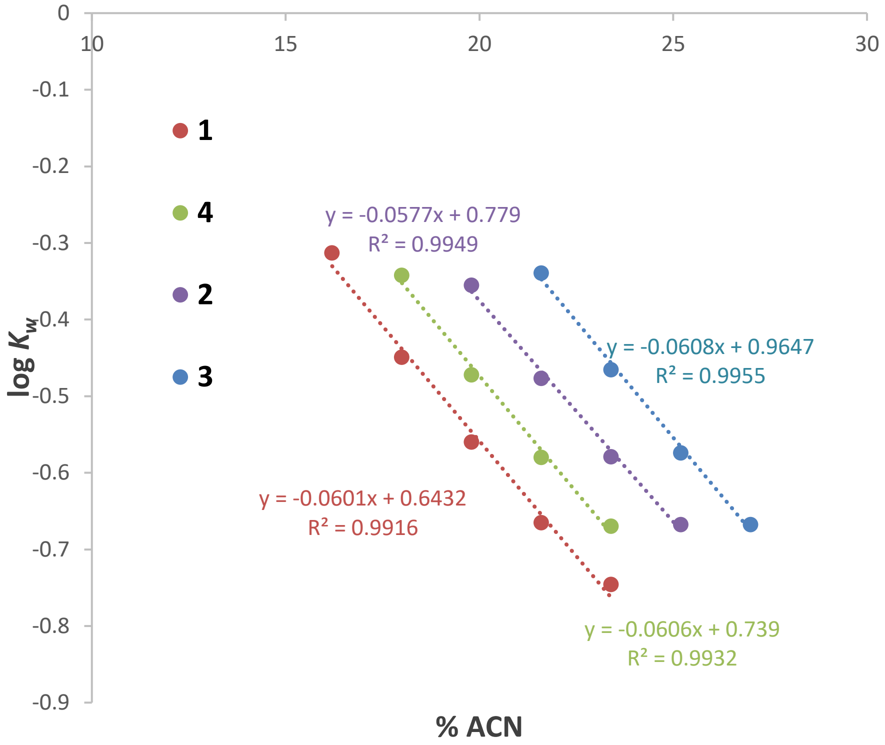

Figure S38. Plot of the concentration of organic solvent vs. $\log k_w$ for **1–4**.

14. In vitro Cytotoxicity Studies

Table S4. The in vitro cytotoxicity values of complexes **1–4**, their platinum(II) and platinum(IV) precursors, biotin, cisplatin, carboplatin and oxaliplatin. GI_{50} values (μM) are reported with standard error of the mean; produced from experiments that were conducted on three separate occasions (n = 3); n.d. = not determined. Resistance factor (RF) defined as GI_{50} in ADDP/GI_{50} in A2780.

	GI_{50} ± SEM (μM)												
**Complex**	**HT29**	**U87**	**MCF-7**	**A2780**	**H460**	**A431**	**Du145**	**BE2-C**	**SJ-G2**	**MIA**	**MCF10A**	**ADDP**	**RF**
**1**	0.17 ± 0.06	0.97 ± 0.15	0.85 ± 0.14	0.25 ± 0.02	0.30 ± 0.02	0.60 ± 0.11	0.12 ± 0.04	1 ± 0.1	0.29 ± 0.07	0.21 ± 0.03	0.28 ± 0.04	0.24 ± 0.01	0.96
**2**	0.011 ± 0.005	0.032 ± 0.008	0.022 ± 0.008	0.020 ± 0.004	0.018 ± 0.004	0.036 ± 0.006	0.004 ± 0.002	0.1 ± 0.01	0.036 ± 0.01	0.010 ± 0.002	0.014 ± 0.004	0.011 ± 0.004	0.55
**3**	0.13 ± 0.05	0.32 ± 0.06	0.099 ± 0.041	0.37 ± 0.03	0.27 ± 0.03	0.82 ± 0.22	0.12 ± 0.04	2.0 ± 0.07	0.57 ± 0.17	0.18 ± 0.01	0.30 ± 0.08	0.17 ± 0.02	0.459
**4**	0.028 ± 0.009	0.11 ± 0.03	0.033 ± 0.004	0.034 ± 0.005	0.032 ± 0.010	0.11 ± 0.02	0.009 ± 0.004	0.1 ± 0.02	0.10 ± 0.03	0.020 ± 0.004	0.031 ± 0.009	0.029 ± 0.005	0.853
**Biotin**	>50	>50	>50	>50	>50	>50	>50	>50	>50	>50	>50	>50	n.d.
**PHENSS(II) [1]**	0.087 ± 0.027	1.2 ± 0.34	0.25 ± 0.041	0.29 ± 0.037	0.30 ± 0.032	0.34 ± 0.10	0.11 ± 0.027	0.39 ± 0.06	0.44 ± 0.055	0.24 ± 0.022	0.24 ± 0.060	0.23 ± 0.049	0.793
**56MESS(II) [1]**	0.015 ± 0.0062	0.076 ± 0.0087	0.033 ± 0.012	0.039 ± 0.0077	0.028 ± 0.0047	0.036 ± 0.011	0.010 ± 0.0013	0.086 ± 0.0030	0.15 ± 0.0067	0.022 ± 0.00088	0.025 ± 0.010	0.027 ± 0.0041	0.692
**47OMESS(II)**	0.12 ± 0.016	0.39 ± 0.06	0.15 ± 0.045	0.23 ± 0.007	0.50 ± 0.058	0.35 ± 0.026	0.085 ± 0.0048	2.6 ± 0.26	1.3 ± 0.067	0.24 ± 0.042	0.43 ± 0.037	0.17 ± 0.010	0.739
**5MESS(II)**	0.033 ± 0.0038	0.32 ± 0.026	0.20 ± 0.012	0.061 ± 0.010	0.041 ± 0.005	0.12 ± 0.025	0.022 ± 0.0027	0.27 ± 0.038	0.22 ± 0.01	0.048 ± 0.002	0.030 ± 0.0018	0.034 ± 0.0023	0.557
**PHENSS(IV) [2]**	0.71 ± 0.30	4.9 ± 0.61	16 ± 4.5	0.80 ± 0.084	1.7 ± 0.20	4.3 ± 0.53	0.31 ± 0.092	3.0 ± 0.53	1.7 ± 0.35	3.4 ± 2.2	1.7 ± 0.20	1.3 ± 0.35	1.625
**56MESS(IV) [1]**	0.036 ± 0.0071	0.19 ± 0.023	0.48 ± 0.14	0.056 ± 0.0071	0.19 ± 0.15	0.12 ± 0.022	0.015 ± 0.0026	0.24 ± 0.022	0.21 ± 0.045	0.043 ± 0.0025	0.061 ± 0.0073	0.17 ± 0.12	3.036
**47OMESS(IV)**	0.52 ± 0.085	2.6 ± 0.39	0.51 ± 0.168	1.7 ± 0.23	3.1 ± 0.21	3.3 ± 0.59	0.27 ± 0.038	33 ± 3.2	7.6 ± 1.8	1.9 ± 0.46	3.7 ± 0.38	1.3 ± 0.058	0.765
**5MESS(IV)**	0.15 ± 0.009	0.83 ± 0.17	0.56 ± 0.200	0.11 ± 0.026	0.18 ± 0.032	0.24 ± 0.007	0.036 ± 0.0032	1.1 ± 0.18	0.60 ± 0.074	0.29 ± 0.034	0.22 ± 0.026	0.18 ± 0.024	1.636
**Cisplatin [1,3]**	11.3 ± 1.9	3.8 ± 1.1	6.5 ± 0.8	1.0 ± 0.1	0.9 ± 0.2	2.4 ± 0.3	1.2 ± 0.1	1.9 ± 0.2	0.4 ± 0.1	7.5 ± 1.3	5.2 ± 0.52	28 ± 1.7	28
**Carboplatin [1,3]**	>50	>50	>50	9.2 ± 2.9	14 ± 1.0	24.3 ± 2.2	14.7 ± 1.2	18.7 ± 1.2	5.7 ± 0.2	>50	>50	>50	n.d.
**Oxaliplatin [1,3]**	0.9 ± 0.2	1.8 ± 0.2	0.5 ± 0.1	0.16 ± 0.0	1.6 ± 0.1	4.1 ± 0.5	2.9 ± 0.4	0.9 ± 0.2	3.0 ± 1.2	0.9 ± 0.2	n.d.	0.822 ± 0.13	5.138

15. Platinum Uptake Studies

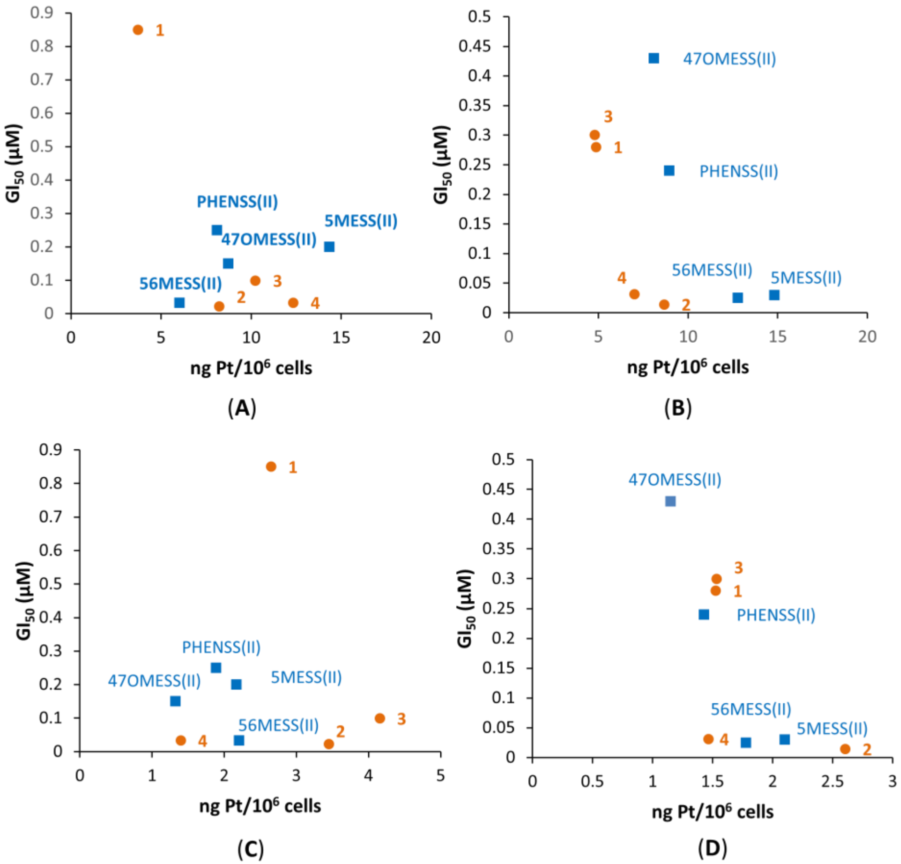

Figure S39. The correlation between the cellular accumulation and cytotoxicity of **1–4** and their platinum(II) precursors against MCF-7 (breast cancer, (**A**) and (**C**)) and MCF10A (normal breast, (**B**) and (**D**)) cells that were treated for 4 hours, at 1.0 µM ((**A**) and (**B**)) and 0.1 µM ((**C**) and (**D**)) concentrations.

16. References

1. Khoury, A.; Sakoff, J.A.; Gilbert, J.; Scott, K.F.; Karan, S.; Gordon, C.P.; Aldrich-Wright, J.R. Cyclooxygenase-inhibiting platinum(IV) prodrugs with potent anticancer activity. *Pharmaceutics*, **2022**, *14*, 787.
2. Deo, K.M.; Sakoff, J.; Gilbert, J.; Zhang, Y.; Wright, J.R.A. Synthesis, characterisation and potent cytotoxicity of unconventional platinum (IV) complexes with modified lipophilicity. *Dalton Transactions*, **2019**, *48*, 17217–17227.
3. Macias, F.J.; Deo, K.M.; Wormell, P.; Clegg, J.K.; Zhang, Y.; Li, F.; Zheng, G.; Sakoff, J.; Gilbert, J.; Aldrich-Wright, J.R. Synthesis and analysis of the structure, diffusion and cytotoxicity of heterocyclic platinum(IV) complexes. *Chemistry a European Journal*, **2015**, *21*, 16990–17001.
